# Genetic and environmental influences interact with age and sex in shaping the human methylome

**DOI:** 10.1038/ncomms11115

**Published:** 2016-03-30

**Authors:** Jenny van Dongen, Michel G. Nivard, Gonneke Willemsen, Jouke-Jan Hottenga, Quinta Helmer, Conor V. Dolan, Erik A. Ehli, Gareth E. Davies, Maarten van Iterson, Charles E. Breeze, Stephan Beck, BIOS Consortium, Peter A.C.'t Hoen, René Pool, Marleen M.J. van Greevenbroek, Coen D.A. Stehouwer, Carla J.H. van der Kallen, Casper G. Schalkwijk, Cisca Wijmenga, Sasha Zhernakova, Ettje F. Tigchelaar, Marian Beekman, Joris Deelen, Diana van Heemst, Jan H. Veldink, Leonard H. van den Berg, Cornelia M. van Duijn, Bert A. Hofman, André G. Uitterlinden, P. Mila Jhamai, Michael Verbiest, Marijn Verkerk, Ruud van der Breggen, Jeroen van Rooij, Nico Lakenberg, Hailiang Mei, Jan Bot, Dasha V. Zhernakova, Peter van't Hof, Patrick Deelen, Irene Nooren, Matthijs Moed, Martijn Vermaat, René Luijk, Marc Jan Bonder, Freerk van Dijk, Michiel van Galen, Wibowo Arindrarto, Szymon M. Kielbasa, Morris A. Swertz, Erik W. van Zwet, Aaron Isaacs, Lude Franke, H. Eka Suchiman, Rick Jansen, Joyce B. van Meurs, Bastiaan T. Heijmans, P. Eline Slagboom, Dorret I. Boomsma

**Affiliations:** 1 Department of Biological Psychology, VU Amsterdam, Van der Boechorststraat 1, 1081BT Amsterdam, The Netherlands; 2 Avera Institute for Human Genetics, 3720 W. 69th Street, Sioux Falls, South Dakota 57108, USA; 3 Department of Molecular Epidemiology, Leiden University Medical Center, Postzone S5-P, Postbus 9600, 2300 RC Leiden, The Netherlands; 4 UCL Cancer Institute, University College London, 72 Huntley Street, London WC1E 6BT, UK; 5 Department of Psychiatry, VU University Medical Center, A.J. Ernststraat 1187, 1081 HL Amsterdam, The Netherlands; 6 Department of Internal Medicine, Erasmus Medical Center, 's Gravendijkwal 230, 3015 CE Rotterdam, The Netherlands; 7 Department of Human Genetics, Leiden University Medical Center, 2300 RC Leiden, The Netherlands; 8 Department of Internal Medicine and School for Cardiovascular Diseases (CARIM), Maastricht University Medical Center, 6200 MD Maastricht, The Netherlands; 9 Department of Genetics, University of Groningen, University Medical Centre Groningen, 9700 RB Groningen, The Netherlands; 10 Department of Gerontology and Geriatrics, Leiden University Medical Center, 2300 RC Leiden, The Netherlands; 11 Department of Neurology, Brain Center Rudolf Magnus, University Medical Center Utrecht, 3508 GA Utrecht, The Netherlands; 12 Department of Genetic Epidemiology, ErasmusMC, 3000 CA Rotterdam, The Netherlands; 13 Department of Epidemiology, ErasmusMC, 3000 CA Rotterdam, The Netherlands; 14 Sequence Analysis Support Core, Leiden University Medical Center, 2300 RC Leiden, The Netherlands; 15 SURFsara, 1090 GP Amsterdam, The Netherlands; 16 Genomics Coordination Center, University Medical Center Groningen, University of Groningen, 9700 RB Groningen, The Netherlands; 17 Medical Statistics Section, Department of Medical Statistics and Bioinformatics, Leiden University Medical Center, 2300 RC Leiden, The Netherlands

## Abstract

The methylome is subject to genetic and environmental effects. Their impact may depend on sex and age, resulting in sex- and age-related physiological variation and disease susceptibility. Here we estimate the total heritability of DNA methylation levels in whole blood and estimate the variance explained by common single nucleotide polymorphisms at 411,169 sites in 2,603 individuals from twin families, to establish a catalogue of between-individual variation in DNA methylation. Heritability estimates vary across the genome (mean=19%) and interaction analyses reveal thousands of sites with sex-specific heritability as well as sites where the environmental variance increases with age. Integration with previously published data illustrates the impact of genome and environment across the lifespan at methylation sites associated with metabolic traits, smoking and ageing. These findings demonstrate that our catalogue holds valuable information on locations in the genome where methylation variation between people may reflect disease-relevant environmental exposures or genetic variation.

## Abstract (web summary)

Differential impact of genetic and environmental influences on DNA methylation may result in sex- and age-related physiological variation and disease susceptibility. By analysing DNA methylome of 2,603 individuals from twin families, here, the authors establish a catalogue of between-individual variation in DNA methylation.

## Graphical abstract

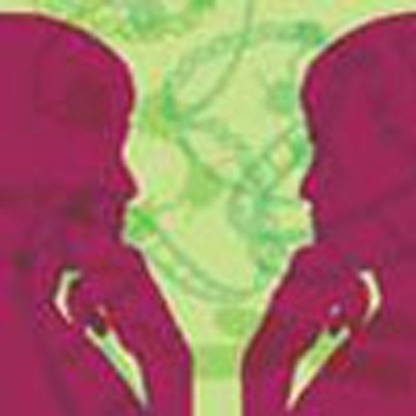

Of the many established epigenetic marks and mechanisms, DNA methylation is thought to contribute to stable long-term gene expression regulation and tissue differentiation^1^, and is ideally suited for genome-wide assessment in large human epidemiological studies^2^. A growing body of literature illustrates that traits and diseases are associated with DNA methylation variation^3^,^4^,^5^,^6^,^7^. DNA methylation differences between individuals may result from differences in environmental exposures^8^, stochastic variation and genetic influences^9^. Increasing evidence suggests that genetically induced epigenetic variation between individuals contributes to human disease susceptibility^5^,^10^,^11^,^12^. Methylation differences have been observed between the sexes^13^,^14^,^15^ and across age^16^,^17^,^18^,^19^, suggesting that epigenetic regulation may also be involved in the widely observed age and sex differences in life history traits and the aetiology of complex diseases^20^,^21^.

It is well-known that genetically identical model organisms such as cloned animals^22^, isogenic plants^23^ and inbred mice^24^ exhibit epigenetic and phenotypic differences. These organisms and human identical twins offer insight into the impact of environmental and stochastic influences on the epigenome. The overall contribution of genetic and environmental differences, from conception onwards, to variation in DNA methylation between humans may be estimated by contrasting the correlation between DNA methylation levels of monozygotic (MZ) and dizygotic (DZ) twins, who share 100% and 50% of segregating genetic variants that contribute to methylation differences, respectively (the classical twin design). Based on previous twin studies, the average heritability of methylation level on cytosine-guanine dinucleotides (CpGs) across the genome has been estimated between 5% and 19% for different tissues^9^,^16^,^25^,^26^, but it is unknown what part can be explained by common genetic variation and to what extent the impact of genetic and environmental influences on DNA methylation depends on sex and age. Studies of humans and rats have described sex-specific effects of prenatal dietary exposures on DNA methylation^27^,^28^ and sex-specific epigenetic effects of *in utero* exposure to an endocrine disruptor have been described in mice^29^. Some studies indicated that certain epigenetic marks including DNA methylation diverge in twin pairs with ageing, suggesting amplification of environmental or stochastic effects on DNA methylation across the lifespan^30^,^31^, although evidence for such effects is not always observed^26^,^32^. A study of neonatal MZ twins reported that twin pairs may show trajectories of divergent, convergent or longitudinally stable methylation patterns after birth^33^. Examples of sites where the relationships between age and DNA methylation depends on genotype^34^ and sex-specific methylation quantitative trait loci (QTL)^35^ have also been reported.

In the current study, we analyse data from a large cohort of twins and family members in whom DNA methylation was measured across the genome (Illumina 450k array) in whole blood. We establish an accurate catalogue (presented in Supplementary Data 1) of between-individual variation in DNA methylation due to environmental effects, total genetic effects and the effects of common genetic variants. We examine differences in epigenetic regulation between the sexes and across age, and test for interactions of genomic effects and environmental effects on methylation with sex and age. Subsequently, we relate our catalogue to previously published loci where DNA methylation is sensitive to smoking, and loci that are epigenetically associated with metabolic phenotypes, including serum metabolite levels, lipid levels and body mass index (BMI). We demonstrate that (1) many smoking-associated CpGs show epigenetic drift (changes in methylation due to deregulated maintenance^36^) with aging; (2) DNA methylation connected to complex traits is characterized by genetically and environmentally induced variation between individuals; and (3) the importance of the environment increases with age at many sites.

## Results

### Heritable and environmental influences on DNA methylation

We characterized the impact of genetic variation and environmental influences on DNA methylation based on genome-wide DNA methylation and SNP data from 2,603 individuals (mean age=37.2, s.d.=13.3; 66% females; Table 1). The study sample included twins (*N*=2,373), parents of twins (*N*=212), siblings of twins (*N*=16) and spouses of twins (*N*=3). DNA methylation data were available for 769 MZ and 424 DZ twin pairs. Methylation levels at 411,169 autosomal sites were analysed. Before analyses, DNA methylation levels were normalized and the normalized methylation *M* values were adjusted for sex, age, measured white blood cell percentages, the first ten principal components (PCs) from the genotype data, methylation array row and sample plate (see Methods section). Of all analysed methylation sites, methylation levels at 7% (*N*=29,783) showed a significant (*α*=1.2 × 10^−7^, gee model, *z*-test using robust estimates) association with sex (Supplementary Fig. 1), and 33% (*N*=135,775) showed a significant (cross-sectional) association with age (Supplementary Fig. 2). White blood cell proportions displayed the expected age-related trend, characterized by a small positive correlation between neutrophil-to-lymphocyte ratios and age (*r*=0.10, *P*=5.2 × 10^−8^, Pearson correlation; Supplementary Fig. 3). Averaged over genome-wide methylation sites, all predictors together explained on average 16% of the variance in DNA methylation between individuals (Supplementary Fig. 4). At most methylation sites, the s.d. of DNA methylation level across individuals was small (Supplementary Fig. 5).

**Figure 1:**
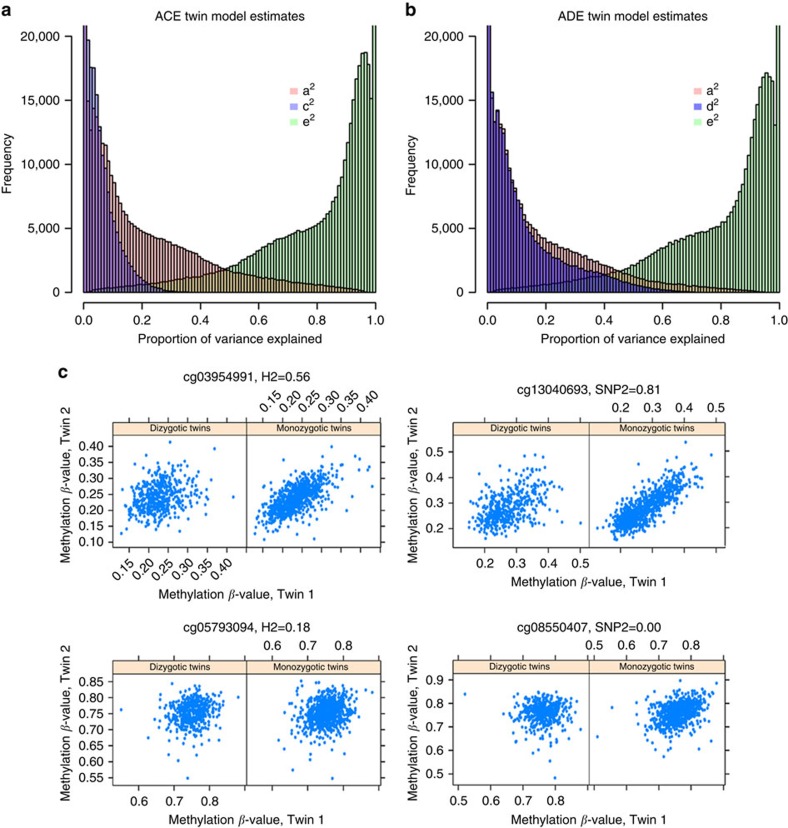
Genetic and environmental influences on genome-wide DNA methylation levels. Estimates from classical twin modelling in 769 MZ and 424 DZ twin pairs. (**a**) Histograms of genome-wide ACE model estimates: variance explained by additive genetic effects (*a*^2^, red), common environmental effects (*c*^2^, purple) and unique environmental effects (*e*^2^, green). (**b**) Histograms of genome-wide ADE model estimates: variance explained by additive genetic effects (*a*^2^, red), non-additive genetic effects (*d*^2^, dark purple) and unique environmental effects (*e*^2^, green). *Y* axes are truncated. (**c**) Scatterplots of DNA methylation levels in MZ and DZ twin pairs at four exemplary CpG sites. The DNA methylation level in twin 2 (*y* axis) is plotted against the DNA methylation level in twin 1 (*x* axis) for all MZ twin pairs and all DZ twin pairs. For illustrative purposes, methylation *β*-values (which represent methylation proportion) obtained after normalization are plotted in this figure, whereas all analyses were performed on normalized methylation *M* values, corrected for a number of covariates (see Methods section). Four examples of CpG sites were selected from the most variable CpG sites (s.d.>0.03) with high heritability (*h*^2^=0.56; upper left), high SNP heritability (*h*^2^_SNPs_=0.81; upper right), low heritability (*h*^2^=0.18; lower left) and low SNP heritability (*h*^2^_SNPs_=0.00, lower right). Note that the larger the difference between the correlation in MZ twins and in DZ twins (stronger correlation in MZ twins), the higher the total heritability. The resemblance of MZ and DZ twins is not informative with respect to the amount of variance explained by genome-wide SNPs. Additional examples are plotted in Supplementary Figs 10–13.

Twin correlations (Table 2) suggested that additive genetic influences mainly account for the resemblance of twins for DNA methylation level; the average correlation in MZ twins (*r*=0.20) is approximately twice as large as the average correlation in DZ twins (*r*=0.09). The data from twins were used to fit classical ACE and ADE twin models that estimate the variance explained by additive genetic effects (A), non-additive genetic effects (D), common environment (C) and unique environmental effects (E) at individual methylation sites (Supplementary Table 1). Note that the variance term E includes stochastic variation and measurement error. ACE models (Fig. 1a) showed that common environmental effects shared by twins explained on average 3% of the variance (s.d.=5%) across all 411,169 methylation sites, with significant C effects (*α*=1.2 × 10^−7^, likelihood ratio test) at 185 methylation sites (0.04%). ADE models (Fig. 1b) showed that non-additive genetics effects explained on average 8% of variance in DNA methylation (s.d.=12%), with significant effects at 241 methylation sites (0.06%). Additive genetic influences had a larger impact on the methylome, with significant effects at 169,013 methylation sites (41%) and accounting for 20% of the variance (s.d.=21%) on average across all methylation sites in the model including only A and E. In the same model, unique environmental effects explained on average 80% of the variance (s.d.=21%).

Next, we estimated the additive genetic and unique environmental variance by fitting linear mixed models in which the variance in DNA methylation was modelled as a function of measured genetic relationships^37^ among 2,603 individuals. To this end, we constructed a genetic relationship matrix (GRM) based on all common genotyped autosomal SNPs (Affymetrix6 array, minor allele frequency (MAF)>0.01). We applied the method of Zaitlen *et al*.^38^, which allows for simultaneous estimation of the total additive heritability of DNA methylation level (hereafter referred to as *h*^2^) and *h*^2^_SNPs_ (variance in DNA methylation level explained by all variants in the genome tagged by genotyped SNPs)^37^ in cohorts that include both closely and distantly related individuals. Because of the limited evidence for common environmental effects and non-additive genetic effects genome-wide, all further analyses focused on additive genetic effects and unique environmental effects.

**Figure 2:**
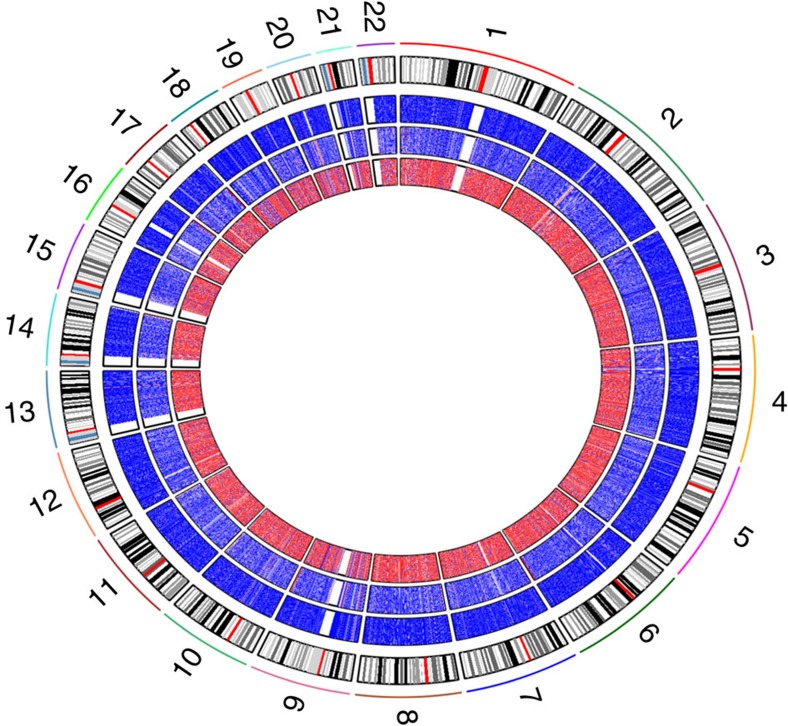
Heritability of DNA methylation level across the genome. Estimates of DNA methylation heritability from the GRM-based approach in 2,603 individuals. From inside to outside: the most inner circular diagram displays the average methylation level at each site, the second band shows the total heritability of DNA methylation level, the third band shows the SNP heritability of DNA methylation level, and the most outer circle shows the chromosome ideograms. Colours range from dark blue (0%) to dark red (100%).

We obtained estimates of *h*^2^ and *h*^2^_SNPs_ at 407,373 sites (98.5%; Fig. 2). The genome-wide average *h*^2^ was 0.19 (s.d.=0.20), similar to the estimate based on the classical twin method (mean *h*^2^_twins_=0.20), and the estimates of *h*^2^ and *h*^2^_twins_ were strongly correlated (*r*=0.99). The results are similar to twin correlations and *h*^2^_twins_ based on Illumina 450k methylation data from peripheral blood published previously based on smaller studies^16^,^25^. Twin correlations and *h*^2^_twins_ were on average larger at methylation sites with a larger variance (Supplementary Table 2). Since annotated SNPs underlying methylation probe binding sites at positions other than the targeted CpG site did not have a substantial effect on the heritability of DNA methylation (Supplementary Fig. 6), we retained these methylation probes in the analyses.

### Variance explained by common genetic variation in the genome

**Figure 3:**
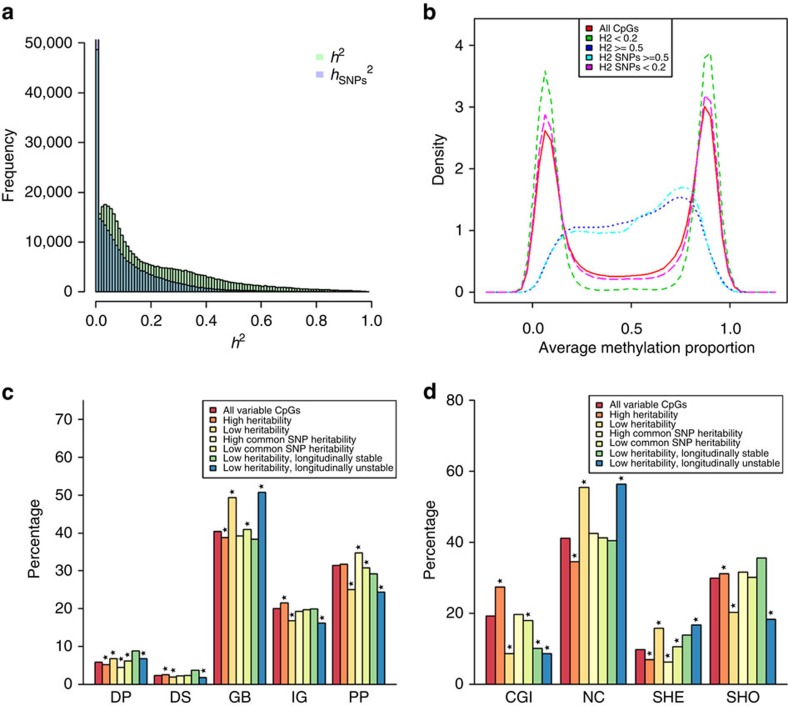
Variance explained by SNPs and longitudinal stability. (**a**) Histogram of total additive heritability (*h*^2^ total) of DNA methylation (green) and variance explained by genome-wide SNPs (*h*^2^ SNPs, purple) for genome-wide CpGs. The *y* axis is truncated. (**b**) Density plot of methylation sites with a high versus low heritability. (**c**) Distribution of methylation sites grouped based on heritability and longitudinal stability relative to genes: DP, distal promoter; DS, downstream region; GB, gene body; IG, intergenic; PP, proximal promoter. (**d**) Distribution of methylation sites grouped based on heritability and longitudinal stability in relation to CpG density: CGI, CpG island; NC, non-CGI; SHE, CpG island shelf; SHO, CpG island shore. Asterisks denote significant enrichment or depletion (*α*=4.27 × 10^−4^, *χ*^2^-test). Results of DNA methylation heritability are from the GRM-based approach in 2,603 individuals. Longitudinal stability was assessed in 31 subjects with data from two longitudinally collected blood samples.

Across all sites, on average 7% (s.d.=12%) of the variance of DNA methylation was explained by common genetic variants in the genome (*h*^2^_SNPs_; Fig. 3a). On average the proportion of total heritability explained by SNPs (*h*^2^_SNPs_/*h*^2^) was 0.37 (s.d.=0.40) (Table 2). At many sites, a relatively large proportion of total estimated heritable variation in DNA methylation was explained by common genetic variants (methylation sites with >0.99 of the total heritability explained by SNPs: *N*=74,226, 18%). However, at many sites DNA methylation is heritable, but common genetic variants explain little of the genetic variance. The SNPs explained <0.01 of the total heritability at 159,299 sites (39%). Moreover, the proportion of variance explained by the unique environmental component (E) was 0.81, highlighting the importance of environmental and stochastic variation.

### Differential heritability patterns across the genome

Methylation sites with high heritability (*h*^2^≥0.5), low heritability (*h*^2^<0.2), high SNP heritability (*h*^2^_SNPs_≥0.5) and low SNP heritability (*h*^2^_SNPs_<0.2) showed different distributions of average methylation level (Fig. 3b) and magnitude of variance across individuals (Supplementary Fig. 7). Sites with a high heritability more often showed intermediate methylation levels and their methylation levels were more variable. Sites with low heritability were usually hypo- or hypermethylated, and their methylation levels were less variable across individuals. We compared sites with a high or low heritability with respect to five gene-centric annotations by mapping them to proximal promoter and distal promoter region, gene body, downstream region and intergenic regions (Supplementary Data 2). We also compared high and low heritable methylation sites with respect to the CpG density of the underlying DNA sequence by mapping CpG islands (CGI), CGI shores, shelves and non-CGI regions (Supplementary Data 2). Across all assessed sites, highly heritable sites showed significant (*α*=4.27 × 10^−4^, *χ*^2^-test) enrichment in CGI shores (*P*<2.2 × 10^−16^, *χ*^2^-test), intergenic regions(*P*<2.2 × 10^−16^, *χ*^2^-test), distal promoter (*P*<2.2 × 10^−6^, *χ*^2^-test) and downstream region (*P*<2.2 × 10^−16^, *χ*^2^-test), which show more variation between people in general, and were depleted in proximal promoter (*P*<2.2 × 10^−16^, *χ*^2^-test), CGIs (*P*<2.2 × 10^−16^, *χ*^2^-test), and shelves (*P*<2.2 × 10^−16^, *χ*^2^-test), which generally display the smallest variation (Supplementary Figs 8 and 9). Importantly, sites with very small variation tend to be biologically invariable, implying that most observed variation may represent technical noise.

To compare highly heritable sites and sites where most variation is stochastic or environmental, we focused on 101,875 methylation sites (25% of all) showing most variation between individuals (standard deviation of the methylation proportion >0.03; hereafter called variable methylated sites), which included 33,329 sites with high heritability, 18,860 sites with low heritability, 5,623 sites with high SNP heritability and 71,788 with low SNP heritability. Exemplary scatterplots of DNA methylation levels in MZ and DZ pairs at sites from each of these categories are provided in Fig. 1c and in Supplementary Figs 10–13. Among variable methylated sites, sites with high heritability and sites with low heritability displayed comparable distributions of total methylation variance (Supplementary Fig. 14). Yet, they showed distinct distributions across genomic regions: Taking all variable methylated sites as reference (Fig. 3c,d), variable methylated sites with low heritability were over-represented in gene bodies (*P*<2.2 × 10^−16^, *χ*^2^-test), distal promoter (*P*=1.5 × 10^−9^, *χ*^2^-test), CGI shelves (*P*<2.2 × 10^−16^, *χ*^2^-test) and non-CGI regions (*P*<2.2 × 10^−16^, *χ*^2^-test), and were underrepresented in proximal promoter (*P*<2.2 × 10^−16^, *χ*^2^-test), intergenic (*P*<2.2 × 10^−16^, *χ*^2^-test), downstream (*P*=1.8 × 10^−4^, *χ*^2^-test), CGIs (*P*<2.2 × 10^−16^, *χ*^2^-test) and shores (*P*<2.2 × 10^−16^, *χ*^2^-test). Variable methylated sites with a low heritability were more often hypermethylated (Supplementary Fig. 15). By contrast, variable methylated sites with a high heritability showed an opposite pattern of enrichment compared with low heritability sites (Fig. 3c,d; Supplementary Data 2), and included many sites with intermediate methylation levels (Supplementary Fig. 15). We also overlaid the most highly heritable and the least heritable variable methylated sites with locations of DNase I hypersensitive sites (DHSs) in 299 individual cellular samples from the Epigenomics Roadmap project^39^. Highly heritable methylation sites showed strong enrichment in DHSs across a number of cell types (Supplementary Data 3), while the least heritable methylation sites were depleted in DHSs of the majority of tissues (Supplementary Data 4). The large majority of probes on the Illumina 450k array target CpG sites, but a small percentage (0.6% of all probes on the array) measure non-CpG methylation. We observed enrichment of non-CpG probes among sites with low heritability (1.7% non-CpG, *P*<2.2 × 10^−16^, *χ*^2^-test, Supplementary Data 5), and depletion among sites with high heritability (0% non-CpG, *P*<2.2 × 10^−16^, *χ*^2^-test).

To further characterize sites with high versus low heritability, we analysed longitudinal peripheral blood DNA methylation data from 31 individuals (mean age 34 years; Table 1) collected with an interval of on average 5 years, and DNA methylation data from blood and buccal samples that were available for 22 individuals (age 18 years; Table 1). At highly heritable sites, the methylation level in blood on average was stable over time (mean *r*=0.73, median=0.76), as previously observed^40^ and correlated weakly on average with methylation level in buccal cells (mean *r*=0.28, median=0.28), whereas sites with a low heritability were not stable (longitudinal correlation: mean *r*=0.08, median *r*=0.08) and did not correlate with methylation level in buccal cells (correlation with buccal: mean *r*=0.00, median *r*=0.00). Thus, genetic influences may underlie stability and cross-tissue correlations for DNA methylation level^41^

Notably, we also observed variable methylated sites with low heritability and high stability across time. Sites that varied mostly due to environmental or stochastic influences and that were longitudinally stable (longitudinal r≥0.5, *N*=542) were significantly underrepresented in CpG islands (*P*<1.0 × 10^−7^, *χ*^2^-test). Longitudinally unstable sites with a large environmental component (longitudinal *r*<0.2, *N*=13,660) were significantly over-represented in shelves (*P*<2.2 × 10^−16^, *χ*^2^-test), non-CGI sites (*P*<2.2 × 10^−16^, *χ*^2^-test), gene bodies (*P*<2.2 × 10^−16^, *χ*^2^-test), and distal promoters (*P*<4.2 × 10^−7^, *χ*^2^-test) and were depleted in CGIs (*P*<2.2 × 10^−16^, *χ*^2^-test), shores (*P*<2.2 × 10^−16^, *χ*^2^-test), intergenic (*P*<2.2 × 10^−16^, *χ*^2^-test), proximal promoter (*P*<2.2 × 10^−16^, *χ*^2^-test) and downstream region (*P*<2.3 × 10^−5^, *χ*^2^-test).

### Genetic and environmental effects vary by sex and age

**Figure 4:**
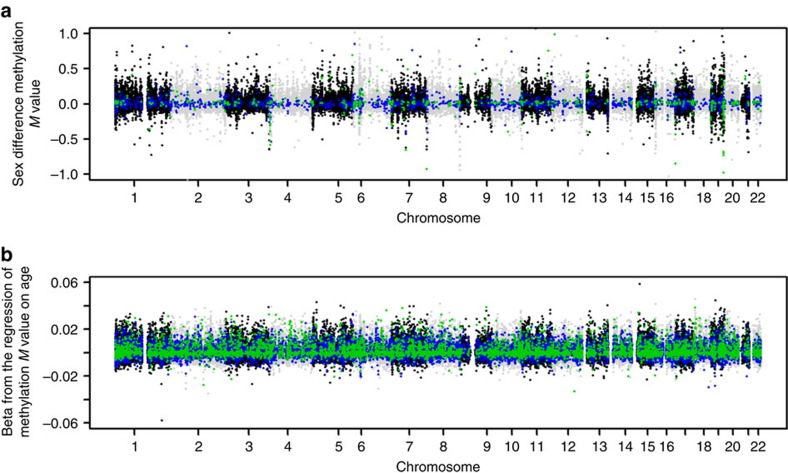
Main effects of age and sex and their interaction with genetic and environmental effects. (**a**) Main and interaction effects of sex. The *x* axis denotes chromosomal position. The *y* axis denotes the *β*-value from the regression of methylation *M* value on sex (while correcting for age, white blood cell counts and technical covariates). Sites where the genetic variance showed significant sex interaction are indicated in blue. Sites where the environmental variance showed significant sex interaction are shown in green. The *y* axis is truncated at −1 and 1 to improve visibility, excluding a small number of sites with regression *β*-values up to −2.4 and 2.6. (**b**) Mean and interaction effects of age. The *x* axis denotes chromosomal position. The *y* axis denotes the *β*-value from the regression of methylation *M* value on age (while correcting for sex, white blood cell counts and technical covariates). Sites where the genetic variance showed significant age interaction are indicated in blue. Sites where the environmental variance showed significant age interaction are shown in green. Results are based on data from 2,603 individuals.

We examined interaction effects between sex and total genetic effects and between sex and unique environmental effects on methylation levels (see Methods section). Sex interaction models were fitted successfully for 391,227 sites (95%). The genome-wide average heritability was nearly identical in males (mean *h*^2^=0.199, median=0.13) and females (mean *h*^2^=0.198, median=0.13). Significant interaction (*α*=1.3 × 10^−7^, *χ*^2^-test) between sex and genetic or environmental effects was evident at 2,667 sites (0.7%; Fig. 4a). At 59% of these sites (that is, 1,572) heritability was lower in women (Supplementary Fig. 16). In a similar manner, we fitted models that included age as a continuous interaction term. Age interaction models were fitted successfully for 379,638 sites (92%). We found significant interaction (*α*=1.3 × 10^−7^, *χ*^2^-test) between age and genetic or environmental effects on DNA methylation at 39,455 sites (10.4%; Fig. 4b). Sex- and age-related differences in heritability may be caused by a difference in the environmental variance or by a difference in the genetic variance. Although both may also occur simultaneously, this is not a general rule. In fact, we found that at 32,234 sites (82%) with significant age interaction, and at 2,034 sites (76%) with significant sex interaction, it was the environmental variance (rather than the additive genetic variance) that was subject to a significant effect of age or sex. This observation highlights that across the genome, environmental or stochastic influences are a more important determinant of sex-specific and age-specific methylation variation between individuals than genetic influences.

**Figure 5:**
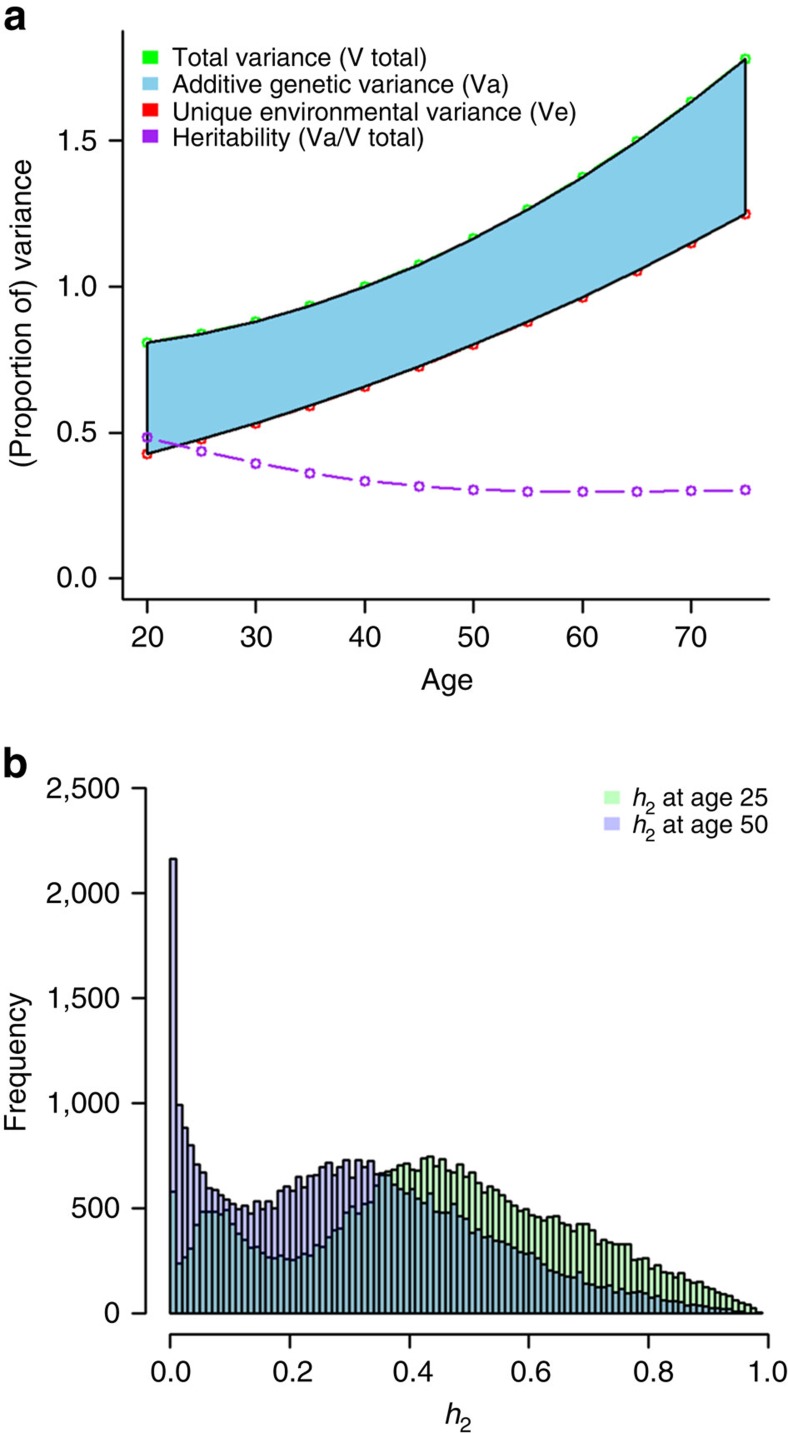
Heritability of DNA methylation at sites with significant interaction between age and genetic variance or age and environmental variance. (**a**) Total variance of DNA methylation, additive genetic variance, unique environmental variance and heritability plotted against age, based on estimates obtained in interaction models on data from 2,603 individuals. (**b**) Histogram of heritability at age 25 (green) and at age 50 (purple) for 39,455 CpGs with significant interaction between age and genetic variance or between age and unique environmental variance. Dark blue denotes the overlap of green and purple bars.

At sites with significant age interaction, the total variance in DNA methylation between individuals generally increases with age, whereas the proportion of variance explained by genetic influences (heritability) decreases, at least up to age 60 (Fig. 5a). While the environmental variance increases at most of these sites across the age range studied, the additive genetic variance initially decreases at most sites, but increases at later ages. At 90% of sites with significant age interaction, the heritability was lower at age 50 than at age 25 (Fig. 5b). At most sites, the change in heritability was modest (Supplementary Fig. 17), but large differences also occurred. For example, there were 104 sites where the change in heritability was larger than 0.5 between age 25 and age 50. Only 21 of these sites were longitudinally stable across 5 years.

### Genomic distribution of sex and age interaction effects

**Figure 6:**
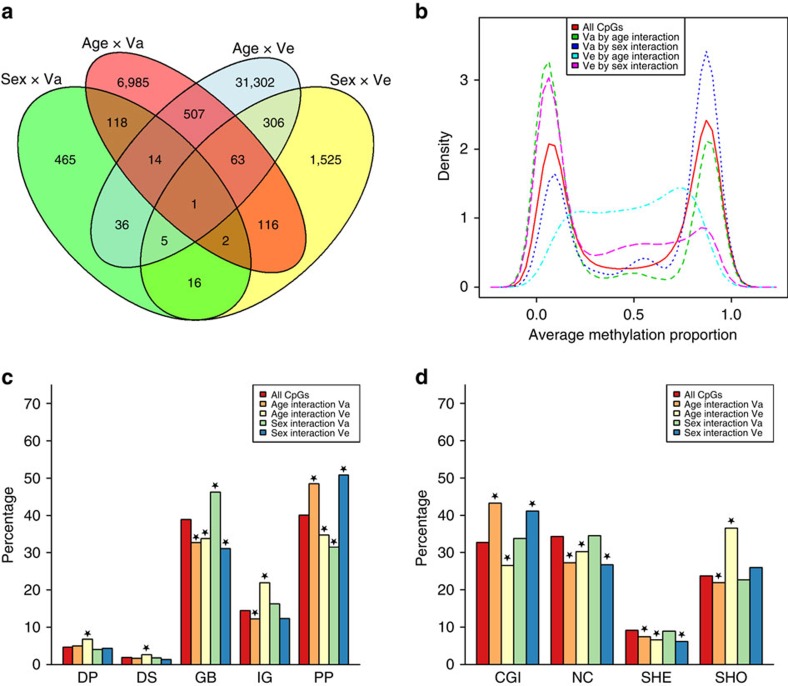
Characteristics and overlap of sites showing interaction between age or sex and genetic or environmental variance. (**a**) Venn diagram of the number of CpGs with significant interaction between sex and genetic variance (Sex × Va), between sex and unique environmental variance (Sex × Ve), between age and genetic variance (Age × Va) and between age and unique environmental variance (Age × Ve). (**b**) Density plot of CpGs with significant interaction effects. (**c**) Distribution of methylation sites with interaction effects relative to genes: DP, distal promoter; DS, downstream region; GB, gene body; IG, intergenic; PP, proximal promoter. (**d**) Distribution of methylation sites with interaction effects in relation to CpG density: CGI, CpG island; NC, non-CGI; SHE, CpG island shelf; SHO, CpG island shore. Asterisks denote significant enrichment or depletion (*α*=4.27 × 10^−4^, *χ*^2^-test). Results are based on data from 2,603 individuals.

While a small proportion of sites shows multiple types of interaction (that is, age and/or sex by genetic and/or environmental influences, Fig. 6a), interactions involving genetic and those involving environmental influences were not equally distributed across genomic sites (Supplementary Data 2). Interactions between environmental effects and age occurred mainly at sites with an intermediate average methylation level (Fig. 6b), and were significantly over-represented (*α*=4.27 × 10^−4^, *χ*^2^-test) in intergenic regions, distal promoters, downstream regions (*P* values <2.2 × 10^−16^, Fig. 6c) and CGI shores (*P*<2.2 × 10^−16^, *χ*^2^-test; Fig. 6d) and underrepresented in gene body, proximal promoter, CGI, non-CGI and shelf (*P* values<2.2 × 10^−16^, *χ*^2^-test). On the other hand, interactions between genetic effects and age occurred more often at hypomethylated sites (Fig. 6b) and were enriched in proximal promoters (*P*<2.2 × 10^−16^, *χ*^2^-test; Fig. 6c) and CGIs (*P*<2.2 × 10^−16^, *χ*^2^-test; Fig. 6d) and depleted in gene body (*P*<2.2 × 10^−16^, *χ*^2^-test), intergenic (*P*=2.2 × 10^−9^, *χ*^2^-test), non-CGI regions (*P*<2.2 × 10^−16^, *χ*^2^-test), shores (*P*=7.2 × 10^−5^, *χ*^2^-test) and shelves (*P*=1.3 × 10^−7^, chi-square test). Sites with sex by environment interaction were usually hypo- or intermediately methylated (Fig. 6b) and were enriched in proximal promoters (*P*<2.2 × 10^−16^, *χ*^2^-test) and CGIs (*P*<2.2 × 10^−16^, *χ*^2^-test), and underrepresented in gene bodies (*P*=3.6 × 10^−13^, *χ*^2^-test), non-CGI (*P*=3.0 × 10^−13^, *χ*^2^-test) and shelves (*P*=2.2 × 10^−6^). Sites with significant interaction between sex and total genetic effects were more often hypermethylated (Fig. 6b), were over-represented in gene bodies (*P*=1.3 × 10^−4^, *χ*^2^-test) and underrepresented in proximal promoters (Fig. 6c, *P*=1.4 × 10^–5^, *χ*^2^-test), and showed no significant differences in distribution relative to CpG density (Fig. 6d). Sites with significant age by genome or sex by genome interaction were not enriched or depleted in DHSs of any cell type from Epigenomics Roadmap data (Supplementary Data 6 and 7). By contrast, sites with significant interaction between environmental effects and age showed significant overlap with DHSs of several types of fetal cells, embryonic stem cells and IPS cells (Supplementary Data 8). Sites with interaction between environmental effects and sex showed enrichment for DHSs across all tissue types (Supplementary Data 9). Interaction between environmental effects and sex on methylation was also enriched among non-CpG sites, while interactions of both genomic and environmental effects with age were depleted among these sites (*P* values< 2.2 × 10^−16^, chi-square test, Supplementary Data 5).

### Some smoking-associated CpGs show epigenetic drift with age

**Figure 7:**
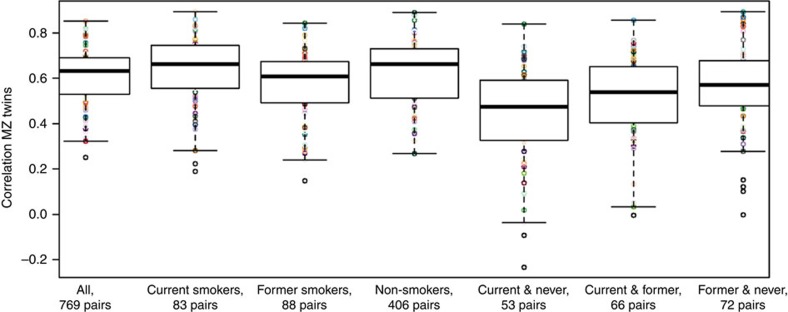
Boxplots of the correlations between DNA methylation levels in smoking-concordant and smoking-discordant MZ twins. Correlations are plotted for DNA methylation level at 65 CpGs that showed significant interaction between age and environmental variance and that were previously found to be associated with smoking^12^. The boxes denote the 25th and 75th percentile (bottom and top of box), and median value (horizontal band inside box). The whiskers indicate the values observed within up to 1.5 times the interquartile range above and below the box.

To further examine the biological relevance, and to gain insight into the causes that may underlie genetically and environmentally induced methylation variance, we compared our findings with genome-wide significant methylation hits from previously published epigenome-wide association studies (EWASs), that is; CpGs where the methylation level in blood is associated with a complex trait or exposure. We first focused on smoking, which has well-replicating associations with DNA methylation level at many CpGs. We examined 430 CpGs associated with current smoking based on the most recently published EWAS^8^. One smoking-associated CpG showed a sex difference in the environmental variance and two smoking-associated CpGs showed a difference in the additive genetic variance (Supplementary Data 10). Comparing smoking-associated locations with 39,455 sites with significant age interaction, overlapping sites included one site that showed age by genome interaction and 65 sites that displayed interaction between environmental effects and age (Supplementary Data 10). Methylation level at cg12803068 in *MYO1G*, associated with smoking and among our top hits for the interaction between age and environment (*P*<1 × 10^−16^, *χ*^2^-test), had a heritability of 0.91 at age 25 and a heritability of 0.71 at age 50. To verify the contribution of smoking to the changing environmental variance with age at all 65 sites, we examined their methylation level in monozygotic twins concordant and discordant for smoking (Fig. 7). Methylation levels were more strongly correlated in smoking-concordant monozygotic twins (concordant current smokers, mean *r*=0.64, concordant never smokers, mean *r*=0.63) than in smoking-discordant twins (discordant for smoking ever, mean *r*=0.44). This observation confirms the role of smoking in the increasing environmental variance with increasing age at these sites. Smoking-associated sites were on average moderately heritable (*h*^2^ mean=0.50, s.d.=0.15), illustrating the presence of both genetic and environmental effects on methylation. It is important to note that smoking itself is a heritable trait.

### Trends in variance at CpGs associated with metabolic traits

We next studied sites where DNA methylation level in blood is associated with metabolic traits, including two CpGs identified by an EWAS meta-analysis of BMI^3^ (*h*^2^=0.72, and *h*^2^=0.88, respectively), eight CpGs associated with lipid levels^6^ (triglycerides, high-density lipoprotein (HDL) or low-density lipoprotein (LDL), *h*^2^ mean=0.45, s.d.=0.12), and 1185 CpGs associated with the levels of a number of distinct serum metabolites^42^ (*h*^2^ mean=0.29, s.d.=0.17). Of metabolite-associated sites, 51 showed age by genome interaction, 70 showed age by environment interaction, one showed sex by genome interaction, and 7 showed sex by environment interaction (Supplementary Data 11). One of the 8 published CpGs associated with lipids showed an interaction effect in our data: at higher ages, unique environmental influences accounted for increasing variation in DNA methylation level at cg22178392 in the *TNIP1* gene, of which DNA methylation level in blood and adipose tissue is associated with serum LDL cholesterol level^6^. The heritability of DNA methylation at this site in blood decreases from 0.54 at age 25 to 0.39 at age 50. Metabolite-associated CpGs displaying a sex difference in the environmental variance include two associated with tryptophan levels, two associated with mannose, and one associated with 5-oxoproline levels (Supplementary Data 11). These point to differences between men and women in the prevalence or impact of exposure to relevant environmental factors that act upon epigenetic regulation of metabolite loci.

### Trends in variance across age at the epigenetic clock

Finally, we examined 353 CpGs included in the epigenetic clock algorithm that predicts DNA methylation age (DNAmAge) across a whole range of tissues^18^. ‘DNA methylation age acceleration' of blood, defined as the difference between DNAmAge and chronological age, was previously found to predict mortality^43^ and to be associated with a number of physical and cognitive fitness measures^44^. Of the clock CpGs, 55 showed interaction between age and genetic or environmental effects (Supplementary Data 12); at 49 clock sites the heritability of DNA methylation level was lower at age 50 compared with age 25, illustrating that environmental and/or stochastic influences account for an increasing portion of the variance between people at higher ages at these sites. This observation is consistent with the finding that the heritability of ‘DNA methylation age acceleration' predicted by these sites is lower in older populations^18^. It also illustrates that there are sites in the genome where the methylation level changes with age, and where there is an age-related shift in the causes of variation between people. This shift generally involves an increase in the impact of environmental or stochastic influences with increasing age. Importantly, our data suggest that this phenomenon not only affects sites where the mean methylation level changes with age (such as the ‘clock CpGs') but also occurs at sites where the average methylation level remains stable across ages (Fig. 4b).

In conclusion, these findings illustrate that our catalogue (Supplementary Data 1) holds valuable information on locations in the genome where methylation variation between people may reflect disease-relevant environmental exposures or genetic variation. Our findings also illustrate that DNA methylation variation at single sites generally shows evidence of both genetic and environmental influences.

## Discussion

We assessed DNA methylation levels in peripheral blood in a large population-based twin cohort, also including family members of twins, and provide a catalogue characterizing the methylation variance of loci along the genome according to genetic and environmental influences and the interaction of these influences with age and sex (Supplementary Data 1). The genome-wide average heritability (*h*^2^) of methylation level was 0.19. Our measured common genetic variants explained on average 7% (*h*^2^_SNPs_) of the methylation variance. Common genetic variants explained on average 37% of the total heritability of methylation level (that is, 0.07/0.19). At 18% of the 450k targeted sites, over 99% of the heritability was explained by common SNPs. Yet, our findings also emphasize that an important part of the heritability of DNA methylation in the genome is not explained by common genetic variants, highlighting the importance of rare variants and structural variants that are not or incompletely tagged by common SNPs on the genotype array.

These findings highlight the importance of environmental and stochastic influences on DNA methylation. Interaction analyses indicated that age and sex-specific heritability of DNA methylation at specific sites is mainly driven by age and sex-specific trends in the environmental variance. In support of previous indications that certain epigenetic marks may diverge between monozygotic twins with age^30^,^31^ (a phenomenon referred to as epigenetic drift), our study revealed a large number of methylation sites where the impact of environmental or stochastic influences on DNA methylation increased with age. Such sites may thus be used to monitor personalized effects of extrinsic and intrinsic factors influencing physiology. We hypothesize that interactions of genetic and environmental effects on DNA methylation with age may be driven by individual differences in intrinsic processes that change with ageing and by accumulating effects of the response to exposures to environmental influences during the lifespan.

Although methylation sites with high and low heritability were observed throughout the genome, comparison of their genomic distribution, taking the most variable sites between people as the reference, revealed that highly heritable sites were enriched, amongst others, in CpG islands and DHSs, while sites where most variation was due to environmental or stochastic influences were depleted in DHSs, CpG islands and proximal promoters and were over-represented in CGI shores, shelves, gene body and distal promoters, especially when longitudinally unstable across ∼5 years. Methylation sites showing significant interaction between environmental effects and age were most strongly enriched in CGI shores. CpG islands and proximal promoters generally show little variation in DNA methylation between people. It is thought that methylation at promoter CpG islands serves a role in long-term repression of genes such as developmental genes and imprinted genes^45^,^46^. By contrast, previous studies have shown that DNA methylation in CGI shores is the most dynamic across tissues and throughout development^47^,^48^,^49^. Our findings suggest that variation in DNA methylation at proximal promoter CGIs as well as DHSs is generally relatively low, leaving genetic differences as the main source for remaining variation between people, while methylation in shores, shelves, non-CGI sites and gene bodies may be more dynamic and more susceptible to environmental or stochastic influences.

This study has several strengths and limitations. Our study is the most comprehensive study to date examining the importance of genetic and environmental influences to individual variation in the human methylome. Yet, this study is limited to DNA methylation measured at a limited number of genome-wide sites in an accessible peripheral tissue. It remains to be examined how representative our findings are for the situation in other tissues, for DNA methylation genome-wide, and for epigenetic marks other than DNA methylation. Although our study included subjects in a very broad age range (17–79 years), it did not cover the entire human lifespan and our interaction analyses were limited to cross-sectional data. Because many human diseases are thought to originate early in life, further studies examining the pre- and postnatal causes of variation in DNA methylation during early development would be extremely valuable. Furthermore, more extensive longitudinal methylation datasets allow better assessment of the genetic and environmental influences on longitudinal stability of methylation levels. By examining sex and age, we considered only a minor subset of medically relevant covariates that may potentially moderate the impact of genetic and environmental influences on DNA methylation. A previous study reported several *in utero* environmental factors that influenced neonate DNA methylation levels in a genotype-specific way, highlighting the importance of genotype by environment interaction^50^. Extension of the interaction model that we used in this study^51^ would allow for the quantification of polygenic gene by environment interaction with measured environmental proxies.

We demonstrated the trends in genetic and environmental variance displayed by sites where DNA methylation level is associated with metabolic traits and smoking, highlighting sites where the environmental or genetic variance of DNA methylation shows differences between males and females or across the life span. Environmental influences on the epigenome may encompass many more types of exposures, including nutrition^52^, exposure to chemicals/pollutants^29^,^53^, stress^54^ and others^55^. In conclusion, we have provided a catalogue (Supplementary Data 1) of genetic and environmental influences on DNA methylation along the genome that can be used to obtain insight into the causes of (sex- and age-specific) variation in DNA methylation at (putative) disease loci.

## Methods

### Subjects and samples

The subjects in this study participated in the Netherlands Twin Register (NTR) biobank project^56^. Venous blood samples were drawn in the morning after an overnight fast, and multiple EDTA and other tubes were collected for isolation of DNA and assessment of haematological profiles. Blood, urine and buccal sample collection procedures were described in detail previously^56^.

The study also included parents of twins, siblings of twins and spouses of twins. In total, 3,264 blood samples from 3,221 NTR participants were assessed for genome-wide methylation, of which 3,089 samples from 3,057 subjects passed quality control. Only samples with good-quality DNA methylation data and with white blood cell counts were retained for analysis, leaving 3,006 samples from 2,975 subjects. This dataset included 769 MZ and 424 DZ pairs. In 31 subjects longitudinal methylation data were available (two time points, mean range=5.2 years, s.d.=1.1, range=2–7 years). All analyses that included genome-wide SNP data were performed on data from a subset of subjects who were genotyped and who were of Dutch origin (*N*=2,603).

For a small subset of 11 MZ pairs (male pairs=3, female pairs=8, age: 18 years), genome-wide methylation data were available for two types of samples: blood (as described above) and buccal. The buccal samples from 10 twins were assessed in 2013, as described by van Dongen *et al*.^57^. The 12 additional buccal samples were assessed using the same protocol in 2014. Buccal and blood samples were collected around the same date.

All subjects provided written informed consent. The study protocols were approved by the Central Ethics Committee on Research Involving Human Subjects of the VU University Medical Centre, Amsterdam, an Institutional Review Board certified by the US Office of Human Research Protections (IRB number IRB-2991 under Federal-wide Assurance-3703; IRB/institute codes, NTR 03-180).

### Cell counts

The following subtypes of white blood cells were counted in blood samples: neutrophils, lymphocytes, monocytes, eosinophils and basophils^56^. Lymphocyte and neutrophil percentages were strongly negatively correlated (*r*=−0.93). Of these two white blood cell subtypes, the percentage of neutrophils showed the strongest correlation with DNA methylation levels (as evidenced by the correlation with PCs from the raw genome-wide methylation data). Basophil percentage showed little variation between subjects, with a large number of subjects having 0% of basophils. Therefore, the percentages of neutrophils, monocytes and eosinophils were used to adjust DNA methylation data for inter-individual variation in white blood cell proportions.

### Genome-wide SNP data

Three distinct genotype data sets were available. The first consisted of previously collected genome-wide SNP data that were only used as part of the quality control (QC) procedure of the DNA methylation data. The second previously collected genome-wide SNP data were used only as part of the statistical analyses of the DNA methylation data. The third SNP dataset consisted of 65 common SNPs targeted by the Illumina 450k array that were only used as part of QC procedure of the DNA methylation data.

#### Genotype data used during QC of the DNA methylation data

Of the 3,221 subjects for whom peripheral blood methylation samples were assessed with the Illumina 450k array, 2,665 subjects had been previously genotyped or had a MZ co-twin who had been genotyped one or multiple times on any of the following genotype arrays: Affymetrix6, Affymetrix-Perlegen and Illumina660. One set of genotypes was selected (the one with the best quality) for MZ twins if both twins were genotyped and for individuals who had been genotyped on multiple platforms. In total, 1,870 genome-wide SNP data sets were available, which were informative for 2,665 individuals (including 795 MZ co-twins). For the DNA methylation data QC, the overlapping SNPs from the Affymetrix6, Affymetrix-Perlegen and Illumina660 arrays were selected. Because of the small overlap of SNPs on these three arrays, this data set was not used for the heritability analyses of DNA methylation.

#### Genotype data used in the heritability analyses

The analyses of DNA methylation heritability were performed using genome-wide SNP data collected with the Affymetrix6 array and SNP data that were extracted from whole-genome sequence data that were available for a small subset of subjects (described previously)^58^. Of the 2,975 subjects with good-quality DNA methylation data and data on white blood cell counts, Affy6 genotype data were available for 2,289 subjects and sequence data for 341 individuals (numbers include both MZ twins). Only SNPs present on the Affy6 platform were extracted from the sequence data. For a subset of 84 subjects for whom sequence data and Affy6 data were available, the sequence data was selected. SNPs with an allele frequency difference between individuals genotyped on Affy6 and individuals who were sequenced were removed (*N*=2,645 SNPs, based on a *P* value<1 × 10^−5^ in a case-control genome-wide association analysis, where case-control status reflected whether a person was genotyped on Affy6 or whole-genome sequenced). The genome-wide SNP data were used to construct a GRM, which summarizes overall genetic relatedness between all subjects (*N*=2603) based on all genotyped autosomal SNPs (MAF>0.01) with genome-wide complex trait analysis (GCTA)^59^.

### Infinium HumanMethylation450 BeadChip data

DNA methylation was assessed with the Infinium HumanMethylation450 BeadChip Kit (Illumina)^60^. Genomic DNA (500 ng) from whole blood was bisulfite treated using the ZymoResearch EZ DNA Methylation kit (Zymo Research Corp, Irvine, CA, USA), following the standard protocol for Illumina 450k micro-arrays, by the department of Molecular Epidemiology from the Leiden University Medical Center (LUMC), The Netherlands. Subsequent steps (that is, sample hybridization, staining, scanning) were performed by the Erasmus Medical Center micro-array facility, Rotterdam, The Netherlands.

### DNA methylation quality control and probe filtering

Quality control and processing of the DNA methylation data from buccal samples has been previously described^57^. The following text describes the quality control and processing of the DNA methylation data from blood samples. The raw intensity files (idat) were imported into the R environment^61^, where further processing, quality control and normalization took place using a protocol developed by the LUMC Molecular Epidemiology department.

First, the methylation data were examined with the R-package MethylAid^62^, which marks outlier samples for a number of quality metrics that are computed based on sample dependent and sample independent quality metrics. The performance of the 3,264 samples is plotted for each of five quality metrics in Supplementary Figs 18–22. Only samples that passed all five quality criteria (using the default MethylAid thresholds) were kept for further analyses. In total, 70 low-performing samples were excluded (2.1%), the majority of which failed based on multiple criteria (Supplementary Table 3). Only the 3,194 samples showing good overall quality were taken on to further processing steps.

Several probe-level QC steps were performed to filter out probes with low performance. For all samples, ambiguously mapped probes were excluded, based on the definition of an overlap of at least 47 bases per probe from Chen *et al*.^63^, and all probes containing a SNP, identified in the Dutch population^58^, within the CpG site (at the C or G position) were excluded, irrespective of minor allele frequency. For each sample individually, probes with an intensity value of zero (not present on the array of a particular sample), probes with a detection *P* value>0.01 (calculated using the function detectionP from the minfi package^64^), and probes with a bead count <3 were excluded. After these steps, probes with a success rate <0.95 across samples were removed from all samples and the success rate across probes for each sample was computed (Mean per sample success rate=99.89%, range=97.86–99.96%). The total number of CpGs after these filtering steps was 421,119. Only autosomal sites were kept in the current analyses (*N*=411,169).

We performed several checks to confirm sample identity, by making use of previously collected genotype data, 65 SNP (control) probes targeted by the Illumina 450k array, and differential methylation patterns in men versus women. Previously collected raw genotype data was used as input for the programme MixupMapper, which computes the probability that a DNA methylation sample matches supplied genotype information based on mQTLs estimated from the dataset^65^. To confirm sex, we clustered samples based on their methylation data, by calculating the Euclidean distance from the pair-wise correlations between samples followed by hierarchical clustering (cluster method=complete linkage). Clustering based on all probes and clustering based on probes in the sex chromosomes yielded similar results. We computed the correlation between samples for 65 SNP (control) probes targeted by the 450 k array to confirm zygosity of twins, and to confirm that longitudinal samples indeed belonged to the same person. Finally, we used the 65 SNP probes to examine potential contamination of samples with foreign DNA, by computing the number of SNPs per sample with an unclear genotype (which we defined as SNPs where the proportion of signal from each allele lay between 0.2 and 0.4 or between 0.6 and 0.8, on a scale from 0 to 1, that is, a pattern not clearly supporting membership to any of the three genotype classes). The number of ‘unclear genotypes' showed a mean of 3.3 across all samples (median=2, s.d.=3.5, Supplementary Fig. 23). We excluded samples with ≥15 unclear genotypes (99th percentile). The genome-wide methylation distribution of these excluded samples showed relatively more intermediate methylation levels (Supplementary Fig. 24). An example scatterplot of the 65 SNP probes in MZ twin samples illustrating DNA contamination of the sample of one of the twins, as detected by this method, is given in Supplementary Fig. 25.

In total, 132 samples were involved in at least one of the following issues: genotype mismatch, sex mismatch, DNA contamination, and inconsistent SNP probe correlation (either between twins or between longitudinal samples from the same person). After solving a swap between two methylation samples identified by MixupMapper (and confirmed by the other checks) by re-swapping methylation data IDs (leaving 128 samples with issues), 67 samples were excluded based on the following grounds: only sex mismatch (22 samples), only genotype mismatch (10 samples), only DNA contamination (27 samples), genotype + sex mismatch (6 samples), DNA contamination + sex mismatch (2 samples). After removal of these samples, there were still 38 samples with an inconsistent SNP probe correlation (that is a zygosity mismatch or mismatch between longitudinal samples), which were all excluded, giving a total of 105 samples (3.3%) excluded based on failed identity or contamination, on top of the 70 samples excluded based on bad quality of the methylation data.

Finally, for 22 persons with Illumina 450k methylation data available from blood and buccal samples, the 65 SNP probes confirmed that blood and buccal samples indeed belonged to the same individual.

### Exploration of technical and biological confounding

To get an impression of the impact of technical and biological effects on overall variation in methylation, principle component analysis (PCA) was performed on the raw genome-wide methylation data (Supplementary Table 4; Supplementary Fig. 26), and the correlation between PC scores and several known technical batches and biological outcomes were computed. PC1 related to sex (*r*=0.92), PC2 was strongly correlated with position on the array (in particular, array row, *r*=0.50), PC3 with several white blood cell counts (for example, lymphocytes: *r*=0.45), and PC4 with age (*r*=−0.59). Other batch variables (for example, 96-well plate, array, and scanner) correlated to a smaller degree with multiple components.

### Methylation data normalization and covariates correction

To reduce technical variability between samples while retaining as much biological variation in DNA methylation as possible, the data were normalized with Functional Normalization, a between-sample normalization method that normalizes the data using PCs (the number of which is user specified) estimated from control probes that are specifically designed not to measure biological variation in samples^66^. There are several strategies to determine the number relevant PCs in a dataset, including inspection of the Eigen values or scree plot, and mathematical algorithms that estimate the number of significant PCs. We chose to perform Functional Normalization with the first 4 PCs, because PCA based on the data from control probes showed that in our data, the first 4 PCs correlated with technical variables (Supplementary Fig. 27), including array row (PC1, *r*=−0.71), scanner (PC2, *r*=−0.46), time (days) between blood sampling and hybridization (PC3, *r*=−0.39), and Illumina 450k array barcode (PC4, *r*=0.18), because the first four PCs had an eigen value >1 (Supplementary Table 5; Supplementary Fig. 28), and because they explained a large proportion of the variance in control probes (89%), whereas each of the further PCs only explained a very small proportion of variance (Supplementary Table 5). We also applied the function EstDimRMT() implemented in the R-package isva, which uses Random Matrix Theory (RMT) to estimate the number of significant PCs^67^, to the control probe data. In convergence with the criteria outlined above, the RMT method retrieved four significant PCs.

Normalized intensity values were converted into beta-values (*β*) and *M* values^11^; *β*-values were used for descriptive purposes only because of their biological interpretability, while *M* values were used as input for all analyses. The *β*-value, which represents the methylation level at a CpG for an individual and ranges from 0 to 1, is calculated as:


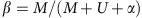


where *M*=Methylated signal, *U*=Unmethylated signal and *α* represents a correction term (100 by default) to control the *β*-value of probes with very low overall signal intensity (that is, probes for which *M*+*U*∼0 after normalization).

The *M* value is equivalent to a log2 logistic transformation of *β*:


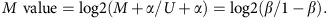


### Genomic annotations

CpGs were mapped to five gene-centric regions: intergenic region (>10 kb from the nearest transcription start site (TSS)), distal promoter (minus(−)10 kb to−1.5 kb from the nearest TSS), proximal promoter (−1.5 kb to plus(+)500 bp from the nearest TSS), gene body (+500 bp to 3′ end of the gene) and downstream region (3′ end to +5 kb from 3′ end), and to CGIs (CG content >50%, length >200 bp and observed/expected ratio of CpGs >0.6), CGI shore (2-kb region flanking CGI), CGI shelf (2-kb region flanking CGI shore) and non-CGI regions, as described by Slieker *et al*^41^. Tests for enrichment among gene-centric and CGI-centric genomic categories were performed with chi-squared tests in R with the function chisq.test() and Bonferroni correction for the total number of performed *χ*^2^-tests (*N* tests=117) was applied to determine statistical significance (*α*=0.05/117=4.27 × 10^−4^). The eFORGE analysis tool (http://eforge.cs.ucl.ac.uk/) was used to test for enrichment among DHSs mapped by the Epigenomics Roadmap Consortium (http://www.ncbi.nlm.nih.gov/epigenomics,^68^), selecting the top 1,000 probes for analyses with >1,000 significant sites. We also annotated previously published CpGs that predict ‘DNA methylation age'^18^, CpGs associated with smoking status^8^, BMI^3^, lipid levels^6^ and metabolite levels^42^.

### Heritable and environmental influences on DNA methylation

To facilitate computations, missing methylation data (0.04–2.14% of genome-wide probes per individual, mean=0.1%) were imputed with the R-package impute. Before analysis, the normalized methylation *M* values were corrected for sex, age, array row, 96-well plate (dummy coded), white blood cell percentages (neutrophils, monocytes and eosinophils; assessed at sample collection), and the first ten PCs derived from the genotype data, with the lm function in R. All analyses that included genome-wide SNP data were performed on the residuals derived after correcting for these covariates. All other analyses (that is, twin correlations and twin models, longitudinal analyses and blood–buccal comparison) were performed on the residuals derived after correcting for the afore mentioned covariates minus the genotype PCs.

The impact of heritable and environmental influences on the methylome was assessed with the classical twin design and with a SNP-based method. Based on the data from twins, a first impression of the classical twin heritability (*h*^2^_twins_) at each methylation site (CpGi) was obtained as follows:


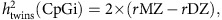


where *r*MZ and *r*DZ are the correlations of DNA methylation level at one CpG site between the MZ, and between the DZ twins, respectively.

Next, genetic models that decomposed variation into additive genetic (A), non-additive genetic (D), common environmental (C) and unique environmental (E) components were fitted to the methylation data of the twins (classical twin method^69^) using maximum likelihood estimation in custom software. The statistical significance of the variance components was evaluated by means of likelihood ratio tests. These models allow estimation of the proportion of variance in DNA methylation attributable to total additive genetic effects (*a*^2^, which represents the same variance component as *h*^2^_twins_), non-additive genetic effects (*d*^2^), common environment (*c*^2^) and unique environment (*e*^2^). The variables *a*^2^, *h*^2^, *c*^2^, *d*^2^ and *e*^2^ represent variance components expressed as a proportion of total variance.

Having established that additive genetic (A) and unique environmental effects (E) are the main sources of variation in the methylome, we proceeded to estimate the proportion of variance attributable to the additive effects of all measured SNPs (*h*^2^_SNPs_), and to test for interactions of total additive genetic effects and environmental effects with age and sex. In these analyses, linear mixed models were fitted in which the covariance of DNA methylation between all individuals (including non-twin family members) was modelled as a function of measured genetic relationships based on SNP data. The approach outlined by Zaitlen *et al*.^38^ was applied, which makes use of two GRMs: a GRM describing the relationships between all individuals (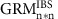) and a second GRM in which all genetic relationships <0.05 IBS (distant genetic relationships) are set to zero (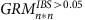), making the estimates of genetic relatedness equivalent to the proportion in the genome shared identity-by-descent (IBD). In essence the covariance between individuals for DNA methylation level at CpGi is modelled as a function of the (very small) genetic covariance between individuals in the population and the (larger) genetic covariance between relatives. Genome-wide SNPs from the Affymetrix6 array (MAF>0.01) were used to construct a GRM with the software programme genome-wide complex trait analysis (GCTA)^59^. For each CpG, we modelled the expected covariance as a function of the GRMs, the additive genetic variance (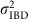), and the variance explained by genome-wide SNPs (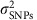) as follows:


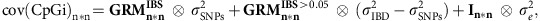


where cov(CpGi)_n*n_ is the expected covariance of DNA methylation at CpGi between individuals, adjusted for covariates, 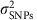 is the variance explained by all SNPs, the term 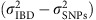 denotes the difference between the total genetic variance and the variance explained by SNPs, and 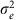 reflects the variance attributable to residual effects (‘unique environment', which may include environmental influences unique to each individual, stochastic influences and measurement error). The total heritability (*h*^2^_IBD_) was calculated as: h^2^_IBD_=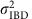/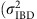
+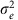). The proportion of variance explained by genome-wide SNPs was calculated as: *h*^2^_SNPs_=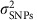/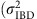
+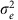) and the proportion of the heritability explained by SNPs was calculated as: *h*^2^_SNPs_/*h*^2^_IBD_.

Genome by sex interaction effects on DNA methylation were investigated with the following model, with sex coded 0/1:


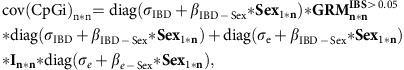


where *β*_IBD-sex_ is the regression coefficient for the interaction of genetic effects with sex, and *β*_*e*-sex_ is the regression coefficient for the interaction of residual effects with sex. These methods are described in detail by Nivard *et al*.^70^ (MGN., Middeldorp C.M., Lubke G., JJH, Abdellaoui A., DIB., CVD. Detection of gene–environment interaction in pedigree data using genome-wide genotypes, under review). This parameterization of the interaction effect is equivalent the method proposed by Purcell^71^.

Genome by age interaction effects on DNA methylation were investigated with the following model, with age *z*-transformed:


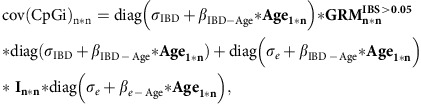


where *β*_IBD-Age_ is the regression coefficient for the interaction of genetic effects with age, and *β*_e-Age_ is the regression coefficient for the interaction of residual effects with age.

Before the analyses based on genome-wide SNP data, methylation levels were standardized (*z*-transformation) to facilitate computations. A small proportion of CpGs for which a model could not be fitted successfully was discarded (see results). The *P* values of each of the four interaction effects (genetic and environmental variance by age and sex) were derived with a *χ*^2^-test (1 degree of freedom), where *χ*^*2*^=(*β*/s.e.)^2^. Statistical significance of interaction *P* values was assessed after Bonferroni correction for the number of CpGs for which estimates were successfully obtained (*α*=0.05/NCpGs; alpha ACE twin modelling=1.2 × 10^−7^, alpha interaction analyses=1.3 × 10^−7^). The correspondence between the classical twin model-based heritability (*a*^2^) and heritability estimated with the GRM approach (*h*^2^) was evaluated by computing the correlation between the value of *a*^2^ and the corresponding value of *h*^2^. Computer code is available upon request from the authors.

### Longitudinal correlation and blood–buccal correlation

Data from individuals with blood samples obtained at two repeated measures were analysed to calculate the correlation between DNA methylation level at time point 1 and DNA methylation level at time point 2 for each CpG site (mean interval=5 years). After obtaining an estimate of heritability and a longitudinal correlation for each CpG, the correlation between genome-wide estimates of (twin-based) heritability and genome-wide estimates of the longitudinal correlation was estimated to examine the relationship between longitudinal stability and the heritability of DNA methylation level. Data from individuals with Illumina 450k methylation data from blood samples and buccal samples were analysed to calculate the correlation between DNA methylation level in blood and buccal for each CpG. Before this analysis, the buccal methylation data (*M* values) were corrected for sex, array row and assessment batch (two levels). Blood–buccal correlations for all CpGs were correlated with *h*^2^_twins_ to examine the relationship between the heritability in blood and the extent to which between-individual variation in DNA methylation level is shared across tissues.

## Additional information

**Accession codes:** The HumanMethylation450 BeadChip data described in this paper are available in the European Genome-phenome Archive (EGA), under the accession code EGAS0001000668.

**How to cite this article:** van Dongen, J. *et al*. Genetic and environmental influences interact with age and sex in shaping the human methylome. *Nat. Commun.* 7:11115 doi: 10.1038/ncomms11115 (2016).

## Supplementary Material

Supplementary Information Supplementary Figures 1-28 and Supplementary Tables 1-5

Supplementary Data 1 Catalogue of genetic and environmental effects on Illumina 450k methylation probes

Supplementary Data 2 Enrichment analysis of heritability results across genomic annotations

Supplementary Data 3 Enrichment test statistics for variable methylation sites with high heritability among DNAse I hypersensitive sites in 299 cellular samples from the Epigenomic Roadmap consortium

Supplementary Data 4 Depletion test statistics for variable methylation sites with low heritability among DNAse I hypersensitive sites in 299 cellular samples from the Epigenomic Roadmap consortium

Supplementary Data 5 Enrichment analysis of heritability results among CpG vs non-GpG probes

Supplementary Data 6 Enrichment and depletion test statistics for sites with significant age by genome interaction among DNAse I hypersensitive sites in 299 cellular samples from the Epigenomic Roadmap consortium

Supplementary Data 7 Enrichment and depletion test statistics for sites with significant sex by genome interaction among DNAse I hypersensitive sites in 299 cellular samples from the Epigenomic Roadmap consortium

Supplementary Data 8 Enrichment test statistics for sites with significant age by environment interaction among DNAse I hypersensitive sites in 299 cellular samples from the Epigenomic Roadmap consortium

Supplementary Data 9 Enrichment test statistics for sites with significant sex by environment interaction among DNAse I hypersensitive sites in 299 cellular samples from the Epigenomic Roadmap consortium

Supplementary Data 10 CpGs previously associated with smoking that showed significant interaction effects

Supplementary Data 11 CpGs previously associated with metabolites that showed significant interaction effects

Supplementary Data 12 Epigenetic clock CpGs' that showed significant interaction effects

## Figures and Tables

**Table 1:** Characteristics of the subjects.

**Sub-group/analysis**	N*	Mean age (s.d.), range	Sex
All subjects heritability analyses	2,603	37.2 (13.3), 17–79	F: 1714, M: 889
MZ twin pairs	769	36.1 (12.4), 18–78	F: 541, M: 228,
DZ twin pairs	424	33.9 (10.5), 17–79	FF: 180, MM: 93, FM: 151
Subjects with longitudinal methylation data	31	34.4 (6.1), 26–50†	F: 24, M: 7
Subjects with blood and buccal methylation data	22‡	18§	F: 16, M: 6

F, female; FF, female-female; FM, female-male; M, male; MM, male-male; MZ, monozygotic; DZ, dizygotic.

^*^Number of subjects or twin pairs.

^†^Age at first blood sample collection.

^‡^Subjects were 11 MZ twin pairs.

^§^All subjects were 18 years when blood and buccal samples were collected.

**Table 2:** Twin correlations, heritability and variance explained by common genetic variants for DNA methylation level at all autosomal CpGs.

**Parameter**	**Min**	**Median**	**Mean**	**Max**
*r*MZ	−0.14	0.12	0.20	0.99
*r*DZ	−0.25	0.06	0.09	0.89
*h*^2^	0.00	0.12	0.19	0.99
*h*_SNPs_^2^	0.00	0.01	0.07	0.98
*h*_SNPs_^2^/*h*_total_^2^	0.00	0.22	0.37	1.00
*h*^2^ Men*	0.00	0.13	0.20	1.00
*h*^2^ Women*	0.00	0.13	0.20	1.00
*h*^2^ Age 25†	0.00	0.14	0.21	0.99
*h*^2^ Age 50†	0.00	0.11	0.18	0.99

*r*MZ, correlation between DNA methylation levels of monozygotic (MZ) twins; *r*DZ, correlation between DNA methylation levels of dizygotic (DZ) twins; *h*^2^, heritability of DNA methylation level; *h*_SNPs_^2^, Proportion of variance of DNA methylation explained by genome-wide common SNPs (MAF >0.01).

^*^Heritability of DNA methylation level in men and women, respectively, as estimated by sex interaction models on all genome-wide autosomal methylation sites.

^†^Heritability of DNA methylation level at age 25 and age 50, respectively, as estimated by age interaction models on all genome-wide autosomal methylation sites.
