# Supplementary material for: Genetic and environmental influences interact with age and sex in shaping the human methylome
Source: Nat Commun. 2016 Mar 30;7:11115. doi: 10.1038/ncomms11115 (PMC4820961; doi:10.1038/ncomms11115)
Supplement: Supplementary Information — Supplementary Figures 1-28 and Supplementary Tables 1-5 [file ncomms11115-s1.pdf]

Supplementary Figures

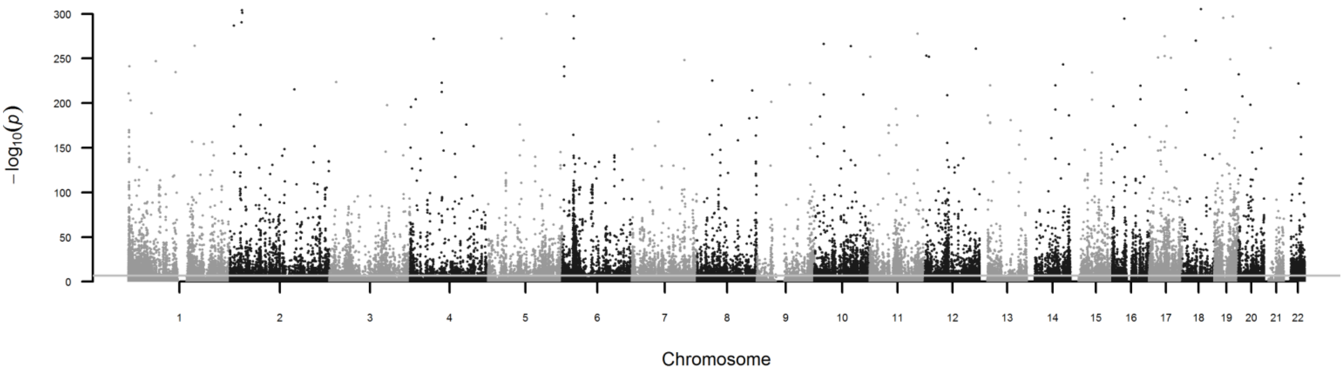

Supplementary Figure 1: Manhattan plot showing the p-values for the association between sex and DNA methylation level plotted against genomic location. P-values for the association between sex and DNA methylation level were obtained using generalized estimation equation models (GEE) with DNA methylation M-value as outcome and the following predictors: sex, age, array row, 96-wells plate (dummy coded), white blood cell percentages (neutrophils, monocytes and eosinophils; assessed at sample collection), and the first ten PCs derived from the genotype data. GEE models were fitted with the R package gee, with the following specifications: Gaussian link function (for continuous data), 100 iterations, and the “exchangeable” option to account for the correlation structure within families. The horizontal grey line represents the genome-wide significant threshold (Bonferroni).

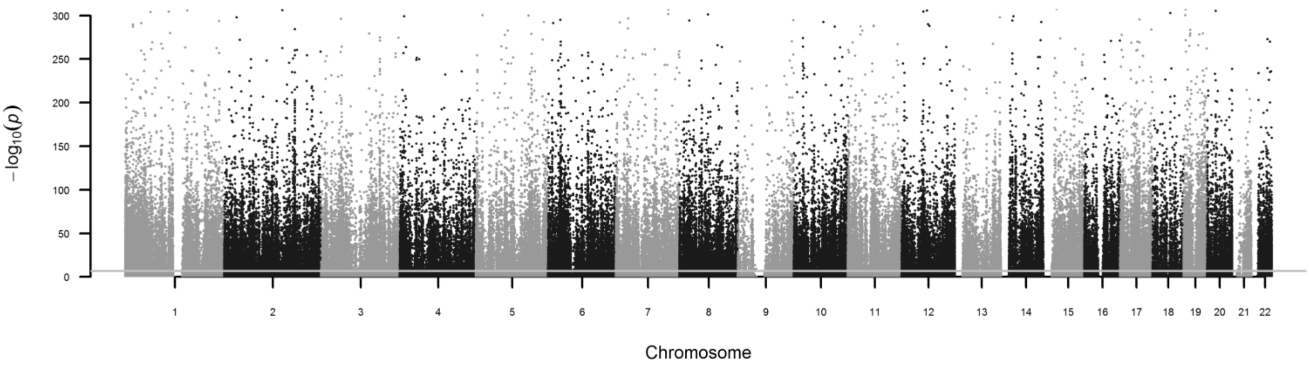

Supplementary Figure 2: Manhattan plot showing the p-values for the association between age and DNA methylation level plotted against genomic location. P-values for the association between sex and DNA methylation level were obtained using generalized estimation equation models (GEE) with DNA methylation M-value as outcome and the following predictors: sex, age, array row, 96-wells plate (dummy coded), white blood cell percentages (neutrophils, monocytes and eosinophils; assessed at sample collection), and the first ten PCs derived from the genotype data. GEE models were fitted with the R package gee, with the following specifications: Gaussian link function (for continuous data), 100 iterations, and the “exchangeable” option to account for the correlation structure within families. The horizontal grey line represents the genome-wide significant threshold (Bonferroni).

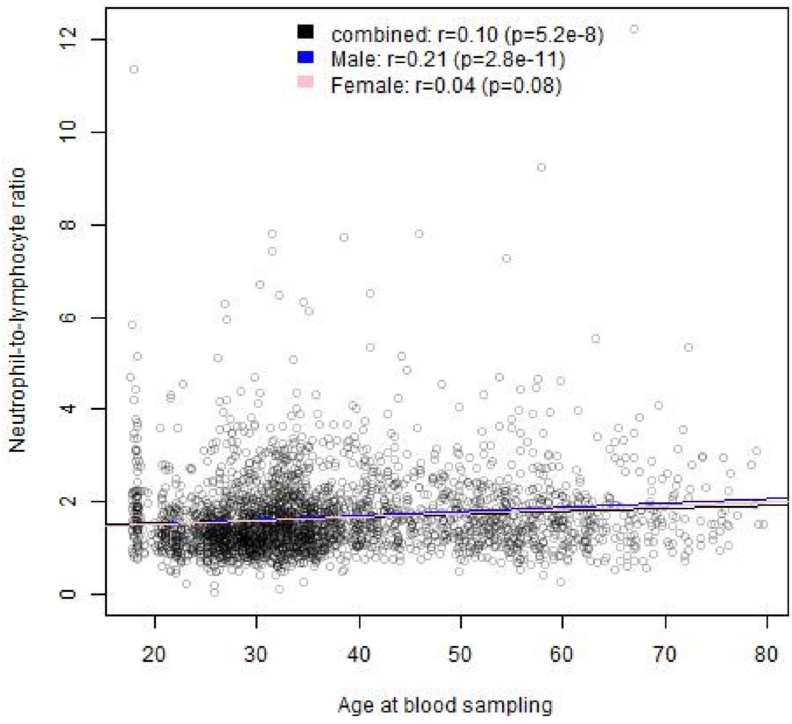

Supplementary Figure 3: Relationship between age and neutrophil-to-lymphocyte ratio. The fitted lines from the linear regression of neutrophil-to-lymphocyte ratio on age in all individuals (black), males (blue) and females (pink) are plotted.

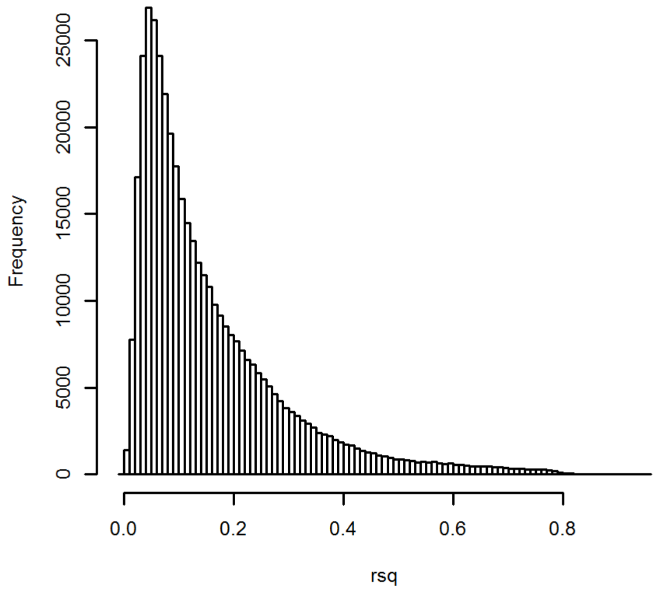

Supplementary Figure 4: Histogram of the variance of DNA methylation level explained by covariates at individual genome-wide CpGs. Rsq=Adjusted r-squared from a linear regression model with DNA methylation level at one CpG site as outcome and the following predictors: sex, age, array row, 96-wells plate (dummy coded), white blood cell percentages (neutrophils, monocytes and eosinophils; assessed at sample collection), and the first ten PCs derived from the genotype data.

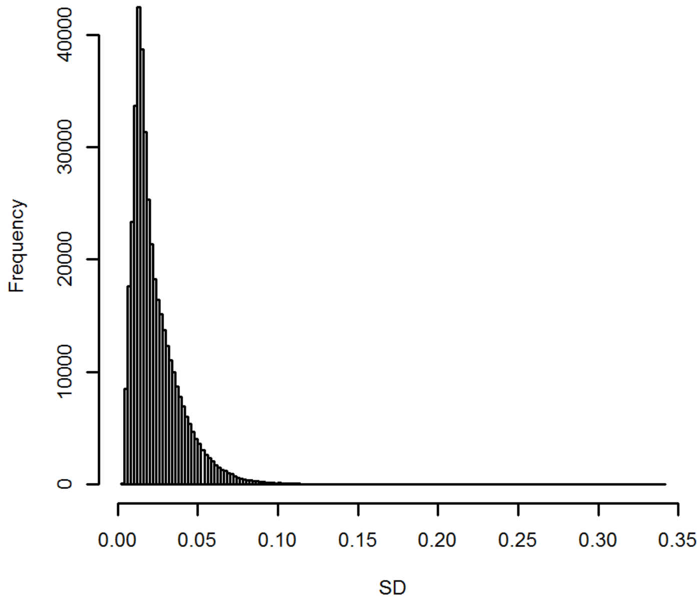

Supplementary Figure 5: Histogram of the standard deviation of the methylation β-value for genome-wide CpGs. SD=Standard Deviation of DNA methylation level (β-value).

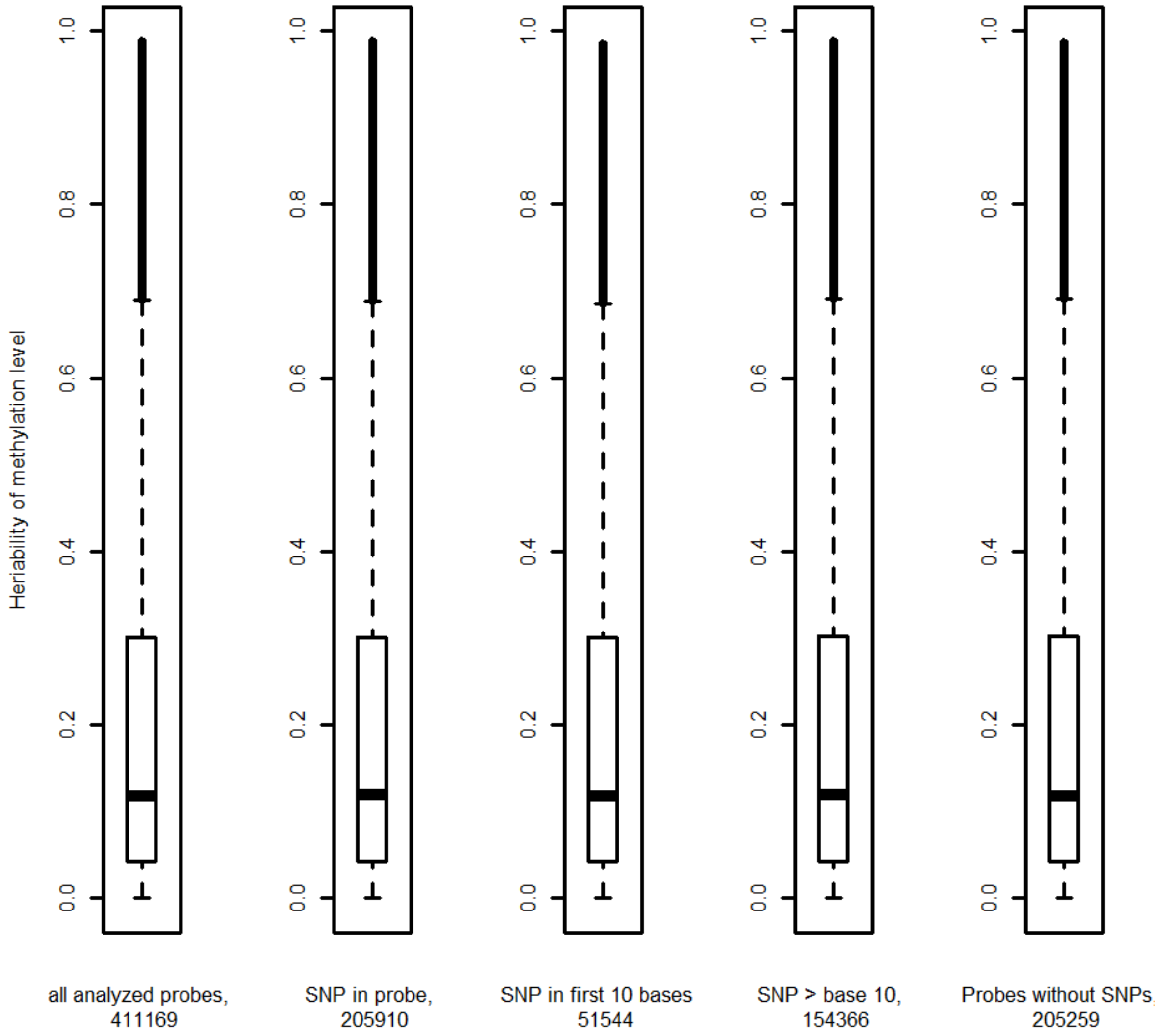

Supplementary Figure 6: Boxplots of the heritability of DNA methylation level in relation to the presence of SNPs within the probe sequence. The first boxplot shows the distribution of heritabilites for all examined autosomal methylation probes. The second boxplot shows the heritabilities of probes containing a SNP anywhere in the probe. The second boxplot shows the heritabilities of probes containing a SNP within the first 10 bases. The third boxplot shows the heritabilities of probes containing a SNP at a position other than the first 10 bases. The last boxplot shows probes without any known SNP in the probe. Probes harboring a SNP within the targeted CpG site were always excluded.

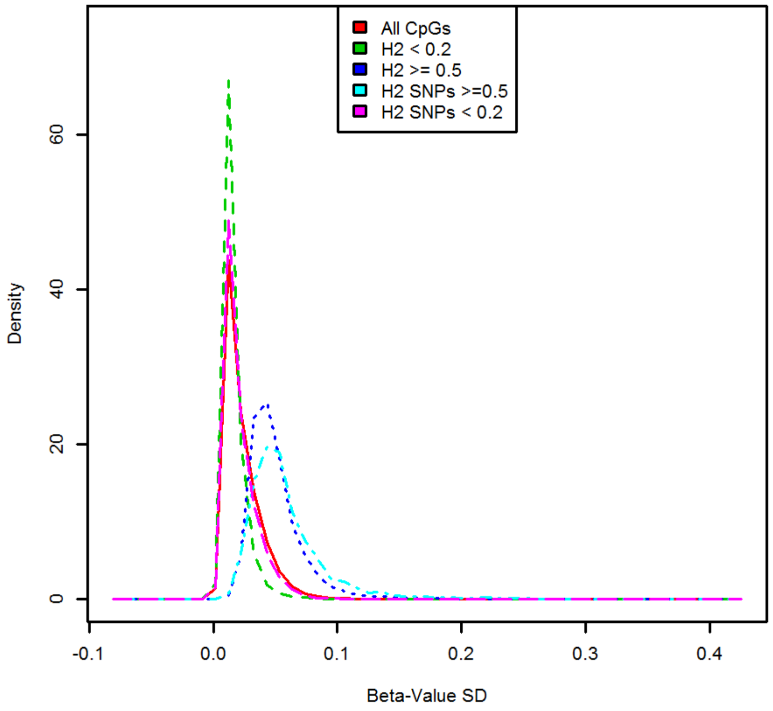

Supplementary Figure 7: Density plot of the standard deviation of the methylation beta-value across individuals for all methylation sites.

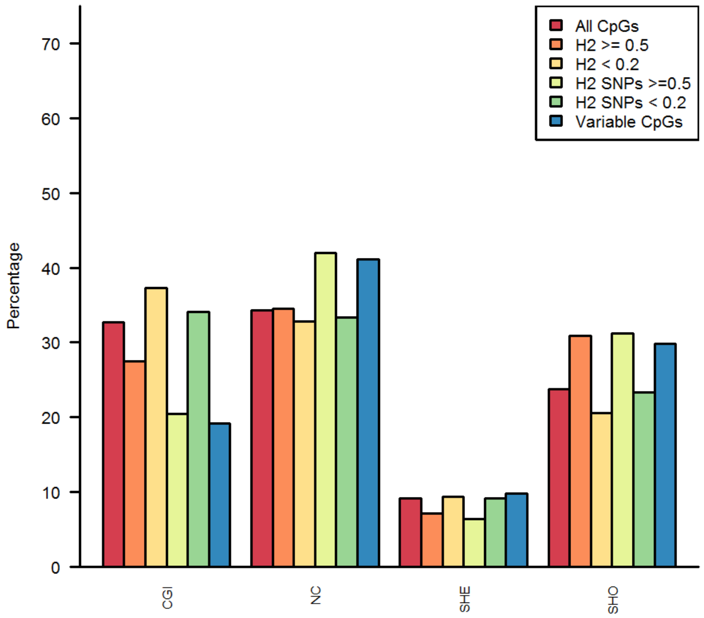

Supplementary Figure 8: Distribution of all methylation sites in relation to CpG density. CGI=CpG island, NC=Non-CGI, SHE=CpG island shelf, SHO= CpG island shore.

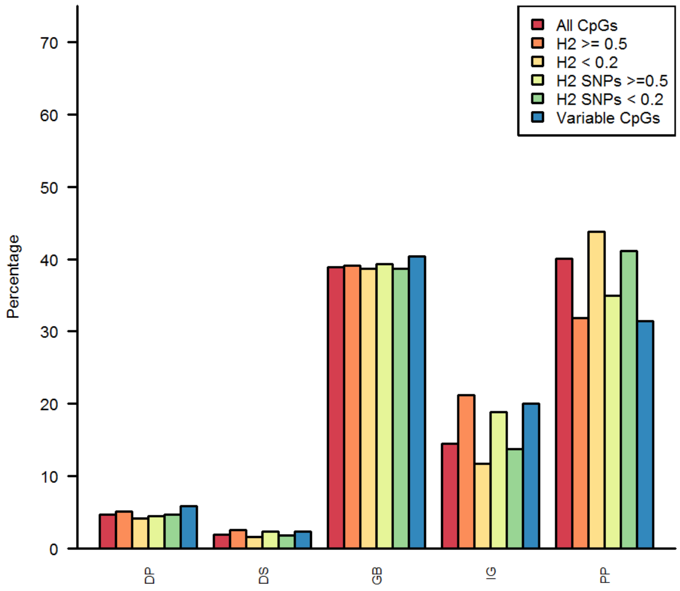

Supplementary Figure 9: Distribution of all methylation sites relative to genes. DP=Distal Promoter, DS=Downstream region, GB=Gene Body, IG=Intergenic, PP=Proximal Promoter.

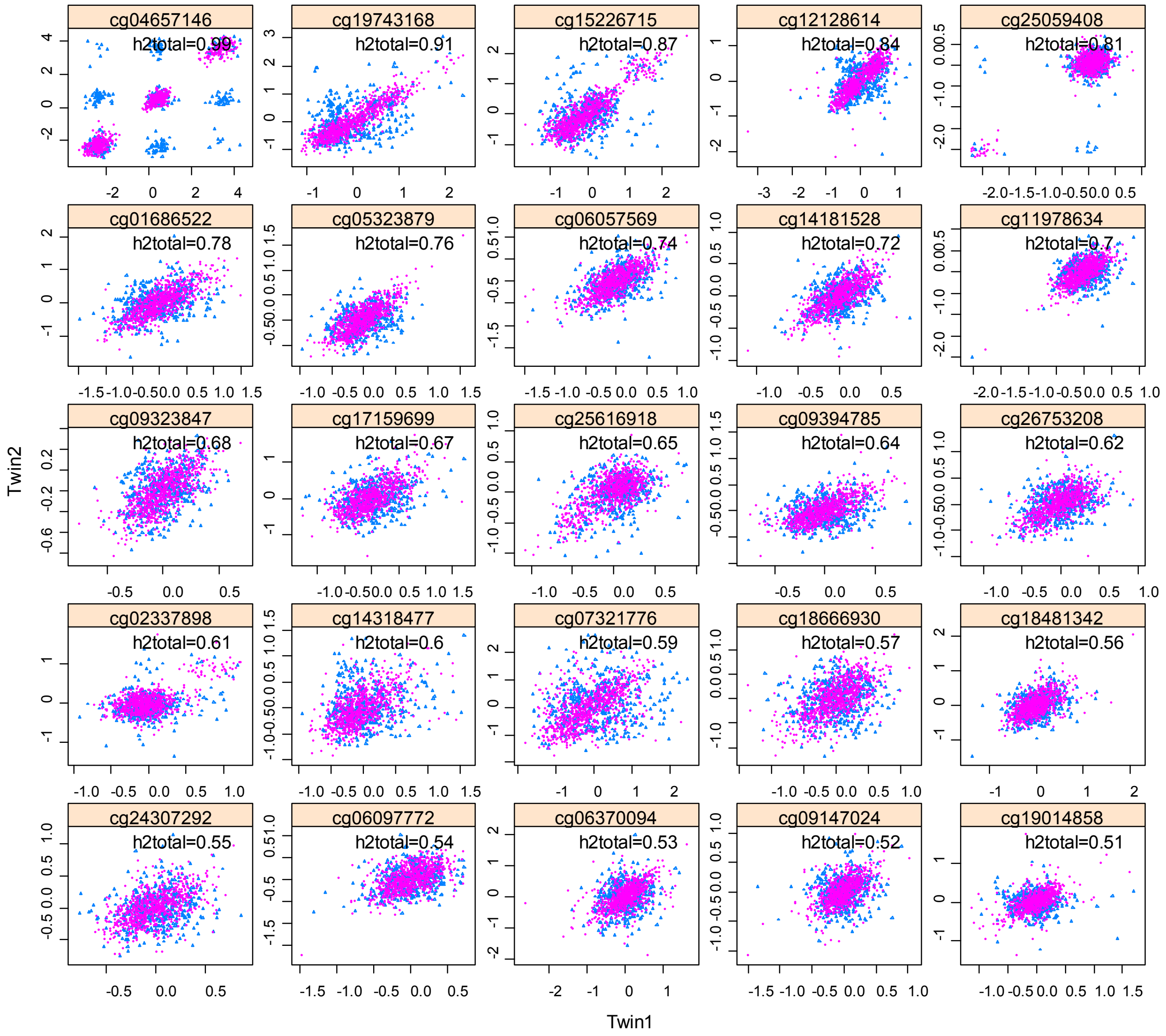

Supplementary Figure 10: Scatterplots of DNA methylation levels in MZ and DZ twin pairs at 25 exemplary CpG sites with high heritability (h^2 >= 0.5). The DNA methylation level in twin 2 (y-axis) is plotted against the DNA methylation level in twin 1 (x-axis) for all MZ twin pairs (pink dots) and all DZ twin pairs (blue triangles). Values represent normalized methylation M-values, corrected for a number of covariates (see methods). CpG sites were selected from the most variable CpG sites (SD > 0.03) with a high heritability (h^2 >= 0.5) by distributing all CpGs in this class into 25 equally-sized bins, and selecting the top site (highest heritability) of each bin. The total heritability of each site is indicated in each plot.

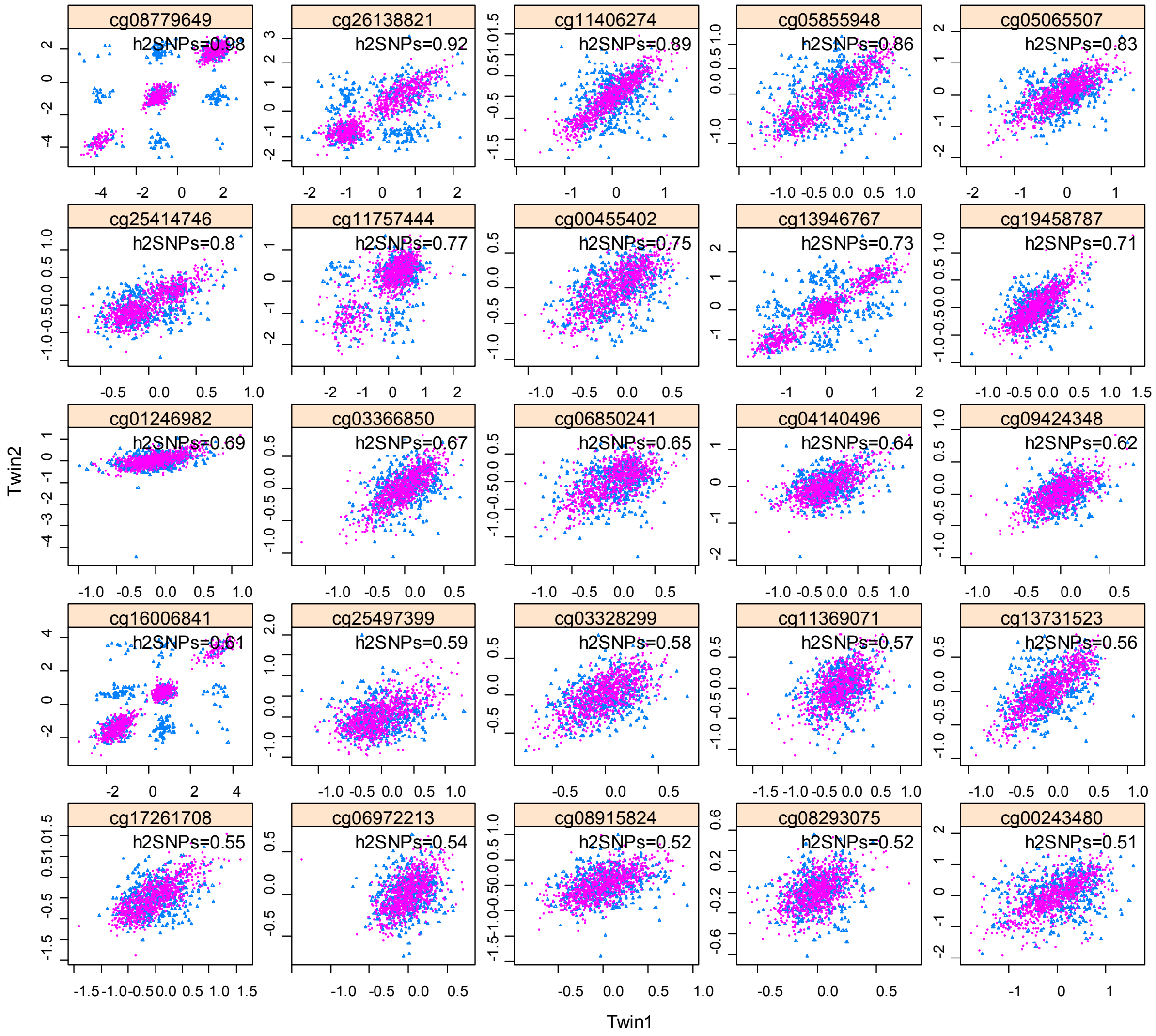

Supplementary Figure 11: Scatterplots of DNA methylation levels in MZ and DZ twin pairs at 25 exemplary CpG sites with high SNP heritability (h^2_{SNPs} >=0.5). The DNA methylation level in twin 2 (y-axis) is plotted against the DNA methylation level in twin 1 (x-axis) for all MZ twin pairs (pink dots) and all DZ twin pairs (blue triangles). Values represent normalized methylation M-values, corrected for a number of covariates (see methods). CpG sites were selected from the most variable CpG sites (SD > 0.03) with a large variance explained by SNPs (h^2_{SNPs} >=0.5) by distributing all CpGs in this class into 25 equally-sized bins, and selecting the top site (highest SNP heritability) of each bin. The proportion of variance explained by SNPs (h^2_{SNPs}) of each site is indicated in each plot.

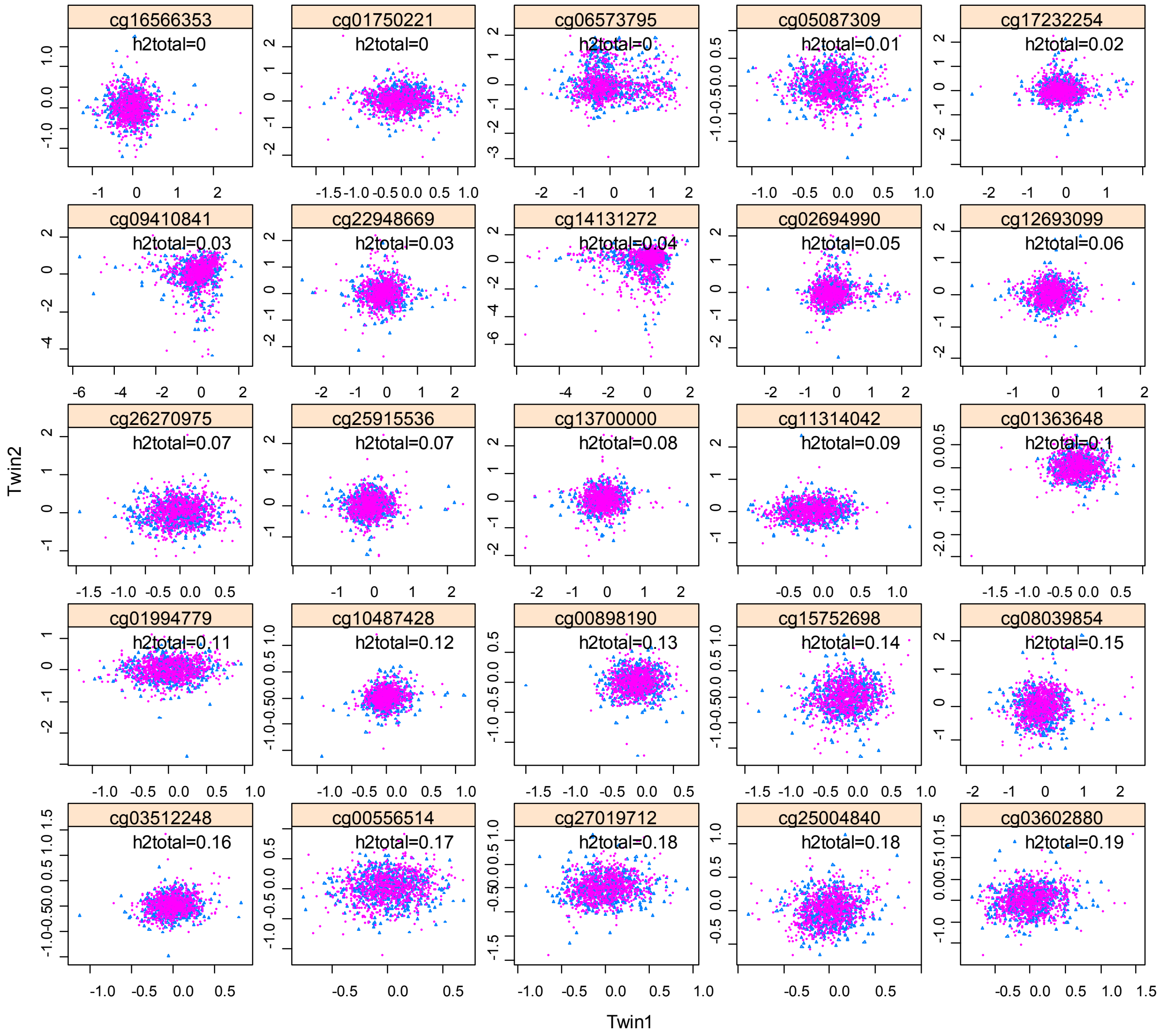

Supplementary Figure 12: Scatterplots of DNA methylation levels in MZ and DZ twin pairs at 25 exemplary CpG sites with a low heritability ($h^2 < 0.2$). The DNA methylation level in twin 2 (y-axis) is plotted against the DNA methylation level in twin 1 (x-axis) for all MZ twin pairs (pink dots) and all DZ twin pairs (blue triangles). Values represent normalized methylation M-values, corrected for a number of covariates (see methods). CpG sites were selected from the most variable CpG sites (SD > 0.03) with a low heritability ($h^2 < 0.2$) by distributing all CpGs in this class into 25 equally-sized bins, and selecting the top site (lowest heritability) of each bin. The total heritability of each site is indicated in each plot.

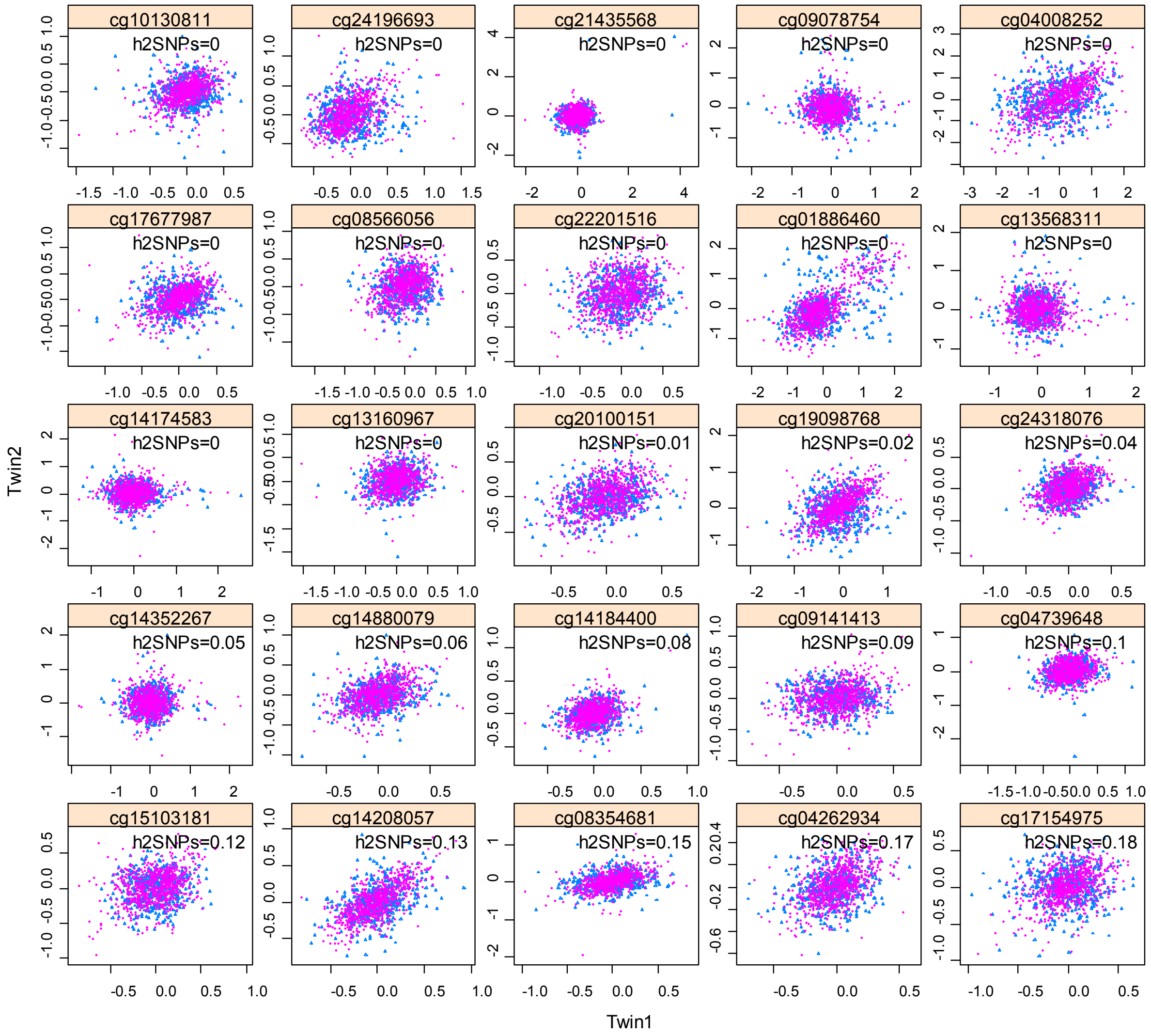

Supplementary Figure 13: Scatterplots of DNA methylation levels in MZ and DZ twin pairs at 25 exemplary CpG sites with a low SNP heritability ($h^2_{SNPs} < 0.2$). The DNA methylation level in twin 2 (y-axis) is plotted against the DNA methylation level in twin 1 (x-axis) for all MZ twin pairs (pink dots) and all DZ twin pairs (blue triangles). Values represent normalized methylation M-values, corrected for a number of covariates (see methods). CpG sites were selected from the most variable CpG sites (SD > 0.03) with a small variance explained by SNPs ($h^2_{SNPs} < 0.2$) by distributing all CpGs in this class into 25 equally-sized bins, and selecting the top site (lowest SNP heritability) of each bin. The proportion of variance explained by SNPs (h^2_{SNPs}) of each site is indicated in each plot.

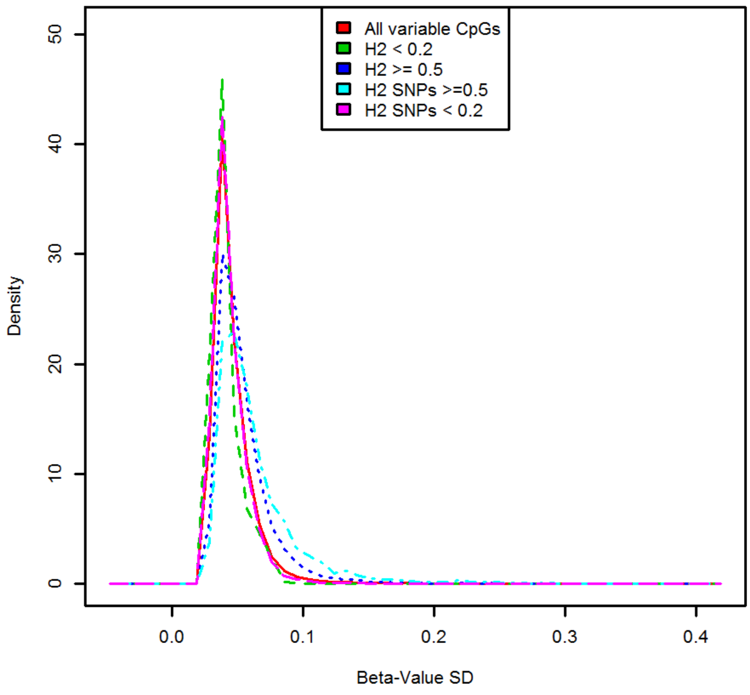

Supplementary Figure 14: Density plot of the standard deviation of the methylation beta-value across individuals for the most variable methylation sites.

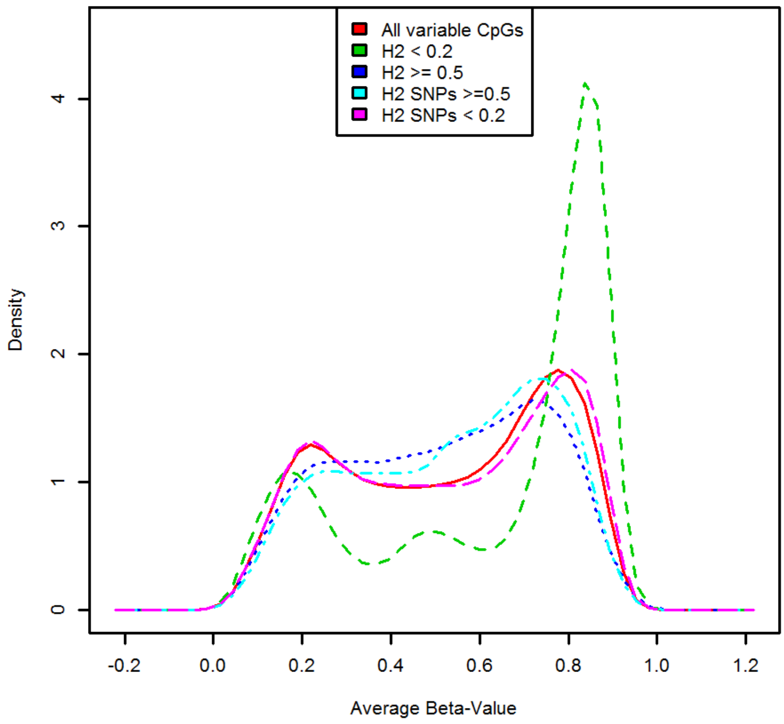

Supplementary Figure 15: Density plot of the methylation beta-value for the most variable methylation sites.

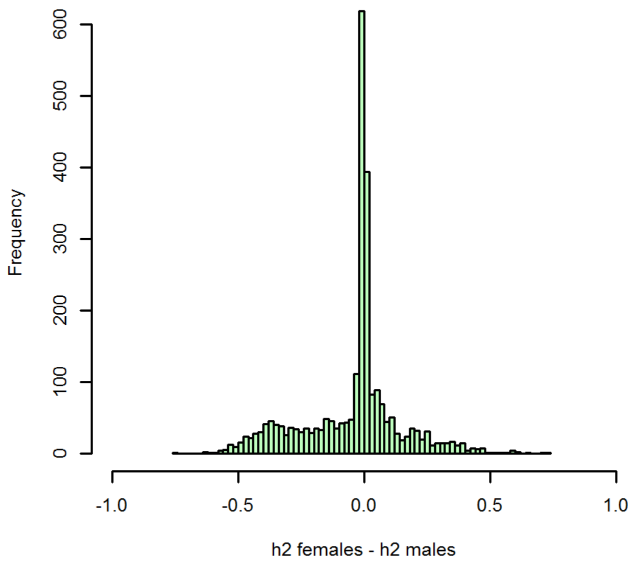

Supplementary Figure 16: Histogram of the difference in heritability between females and males. h^2 in females minus h^2 in males is plotted for 2667 CpGs with significant interaction between sex and genetic variance or between sex and unique environmental variance.

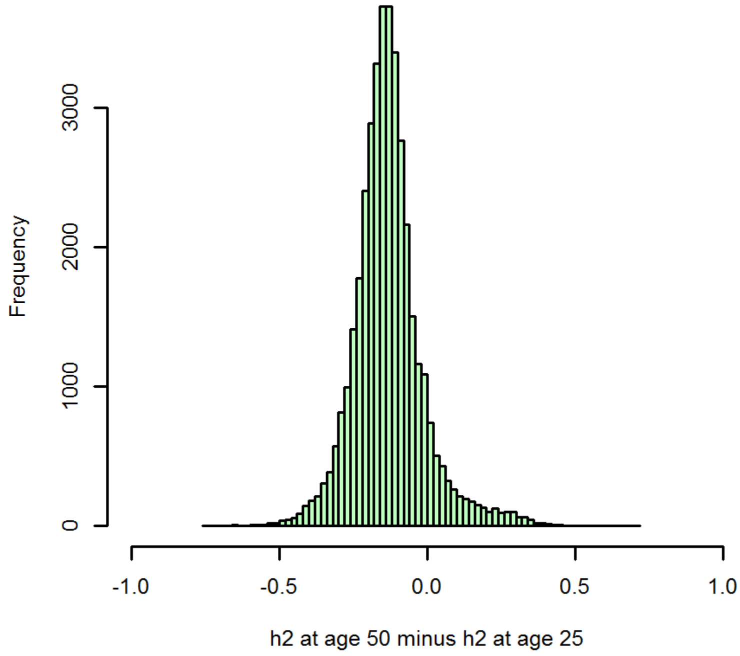

Supplementary Figure 17: Histogram of the difference in (IBD-based) heritability between age 50 and age 25. h^2 at age 50 minus h^2 at age 25 is plotted for 39455 CpGs with significant interaction between age and genetic variance or between age and unique environmental variance.

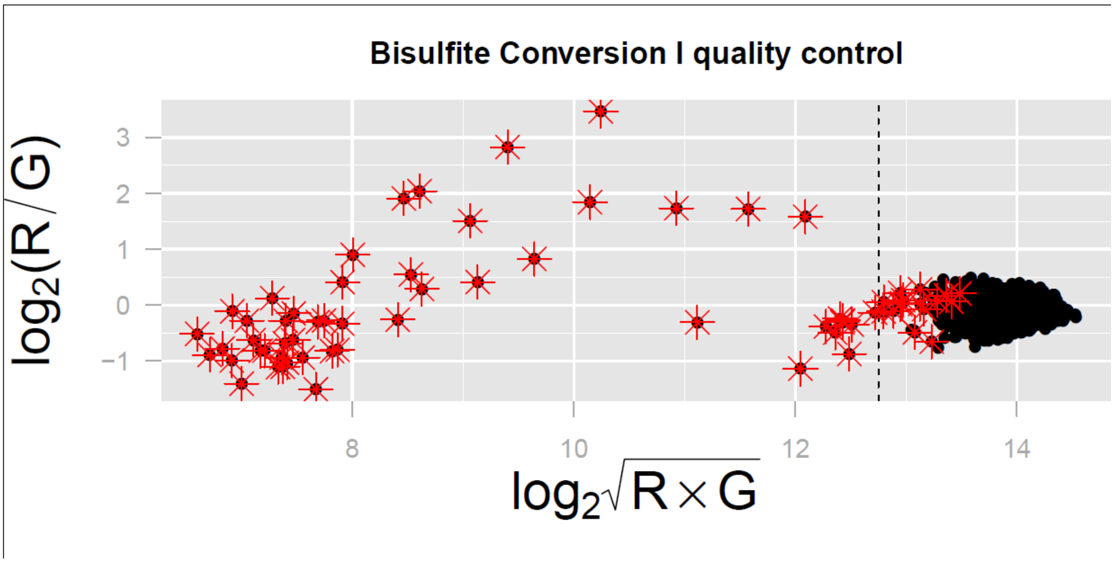

Supplementary Figure 18: Quality control plot of bisulfite conversion. The performance of bisulfite conversion quality control probes is plotted for all DNA methylation samples. Red stars denote samples that failed on the basis of any of the five quality metrics. R=Red Channel. G=Green Channel.

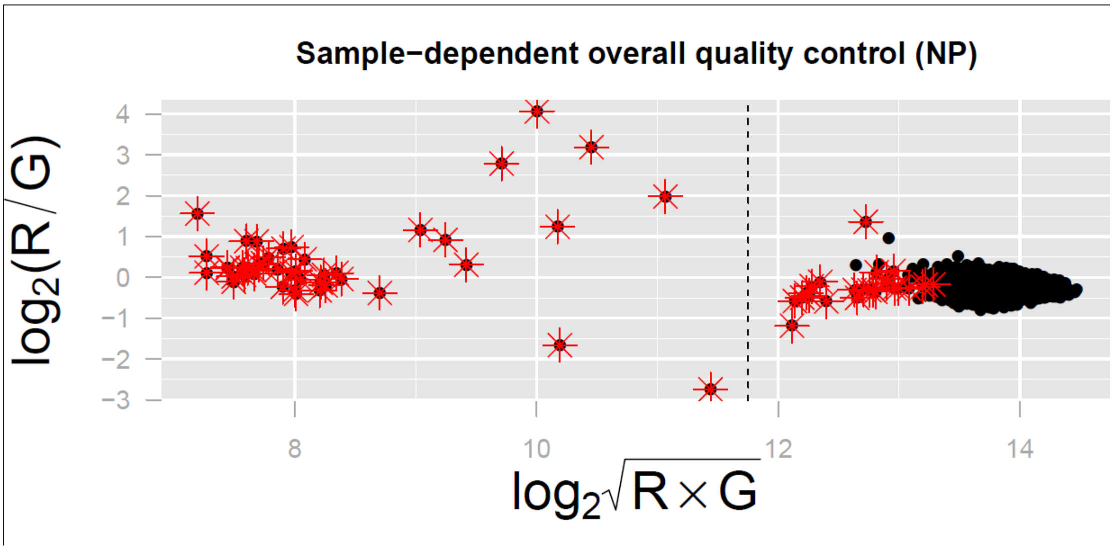

Supplementary Figure 19: Quality control plot of overall sample quality based on sample-dependent control probes (Non-Polymorphic quality control probes). The performance of Non-Polymorphic quality control probes is plotted for all DNA methylation samples. Red stars denote samples that failed on the basis of any of the five quality metrics. R=Red Channel. G=Green Channel.

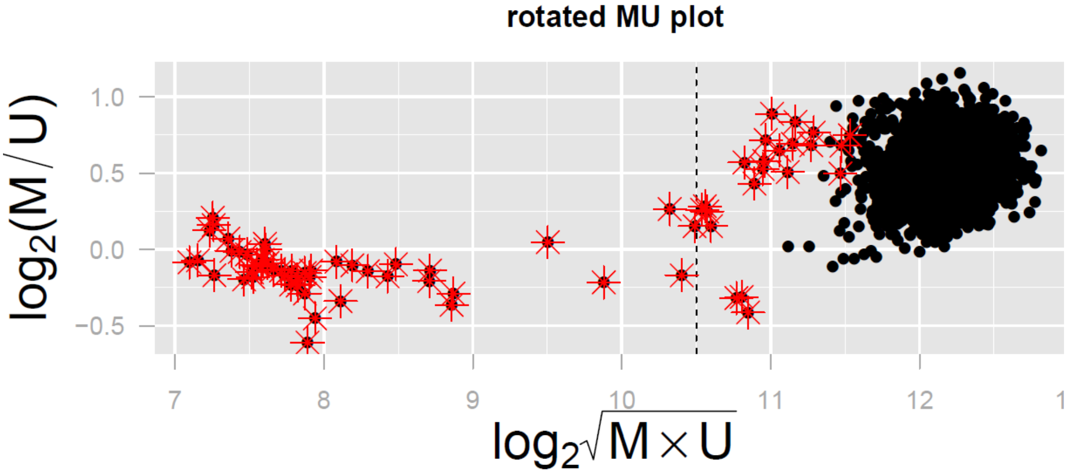

Supplementary Figure 20: Quality control plot of the median Methylated versus Unmethylated signal intensity. The relationship between the Median Methylated (M) and Unmethylated (U) signal intensity is plotted for all DNA methylation samples. Red stars denote samples that failed on the basis of any of the five quality metrics.

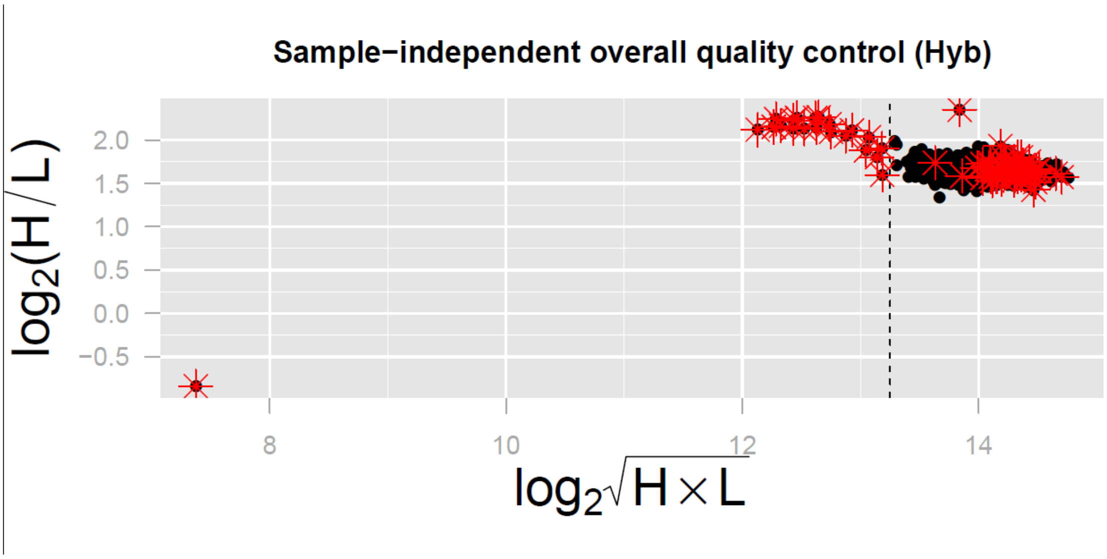

Supplementary Figure 21: Quality control plot based on sample-independent hybridization control probes. The performance of sample-independent hybridization control probes is plotted for all DNA methylation samples. Red stars denote samples that failed on the basis of any of the five quality metrics.

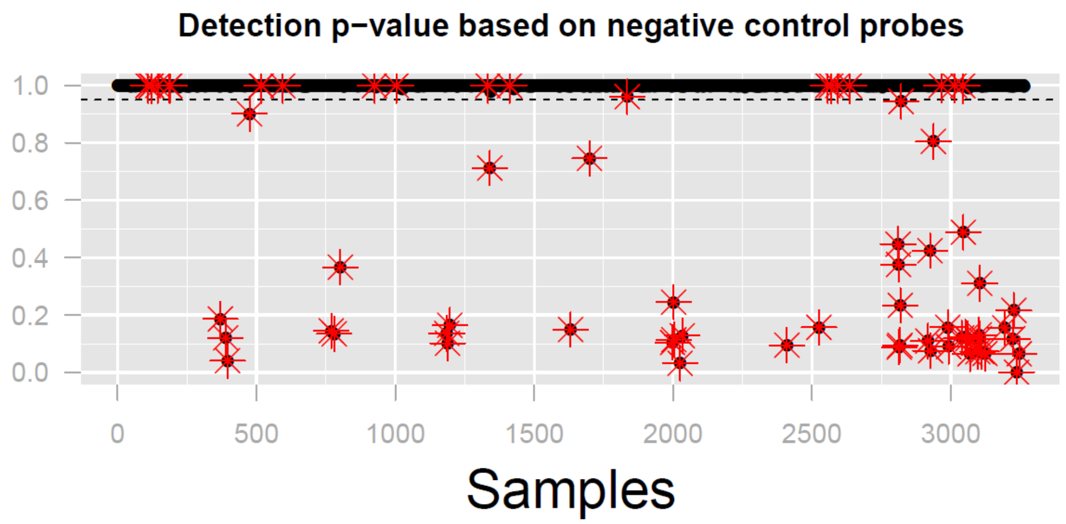

Supplementary Figure 22: Quality control plot showing the proportion of probes with a detection p-value < 0.01 within samples. For all methylation samples, the proportion of probes per sample with a detection p-value < 0.01 is plotted (y-axis). The detection p-value indicates whether the probe signal exceeds the background signal, where the background signal is calculated using the negative control probes. Red stars denote samples that failed on the basis of any of the five quality metrics.

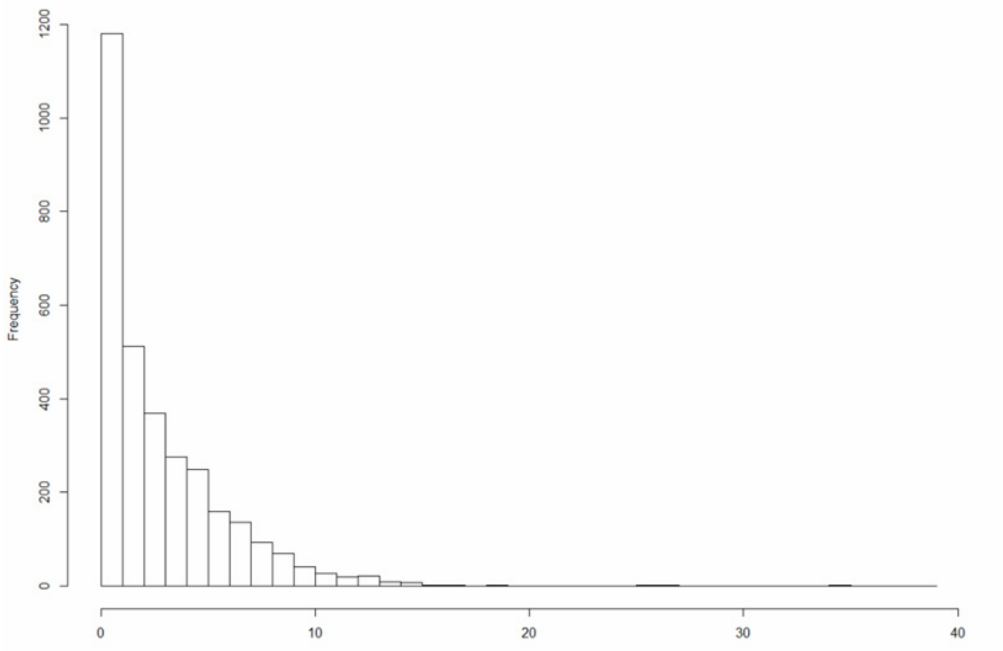

Supplementary Figure 23: Histogram of the number of Illumina 450k SNP probes per sample displaying an unclear genotype. X-axis= the number of unclear genotype per sample: SNPs where the proportion of signal from each allele lay between 0.2 and 0.4 or between 0.6 and 0.8, on a scale from o to 1, i.e. a pattern not clearly supporting membership to any of the three genotype classes. In total 65 common SNPs from the Illumina 450k array were assessed. Y-axis=Number of methylation samples. Methylation samples with >= 15 unclear genotypes (99th percentile) were excluded from analyses.

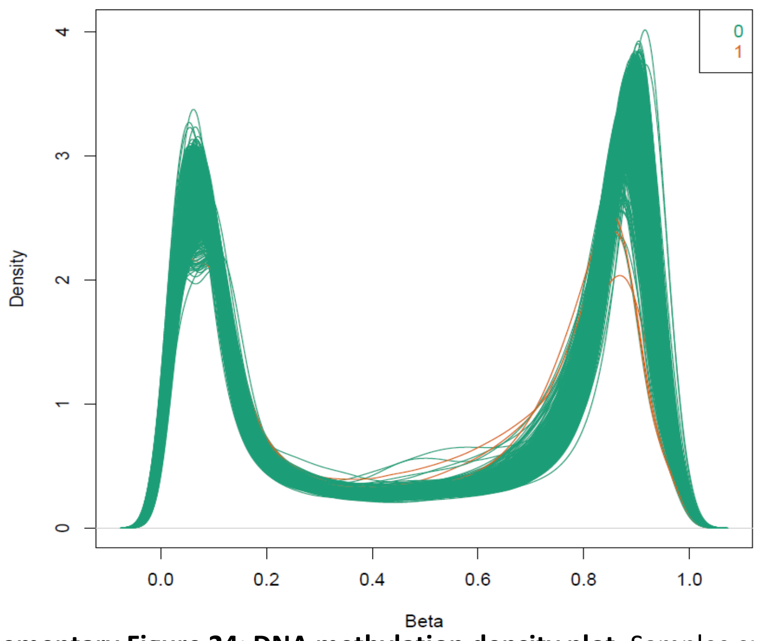

Supplementary Figure 24: DNA methylation density plot. Samples excluded based on suspected DNA contamination are in orange (1) and all other samples are plotted in green (0).

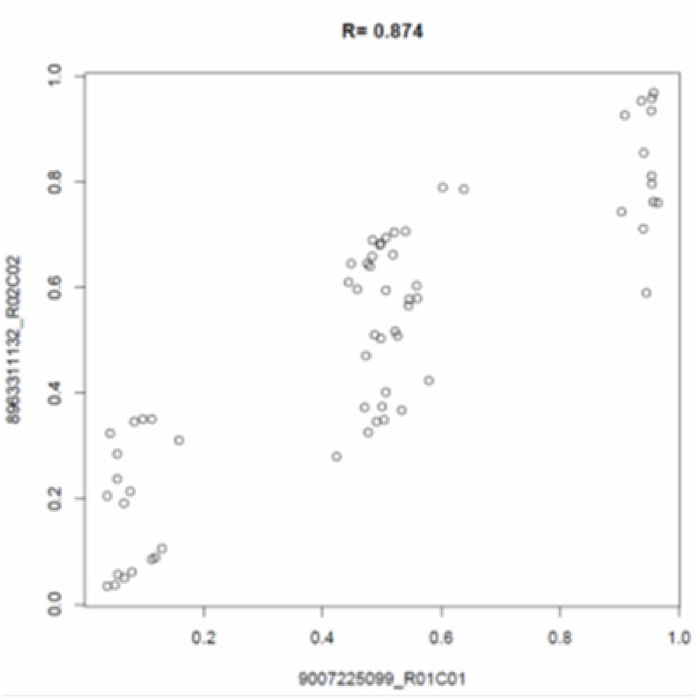

Supplementary Figure 25: Example scatterplot of the 65 Illumina 450k SNP probes in one pair of MZ twins, of which one twin (on the y-axis) was excluded based on suspected DNA contamination.

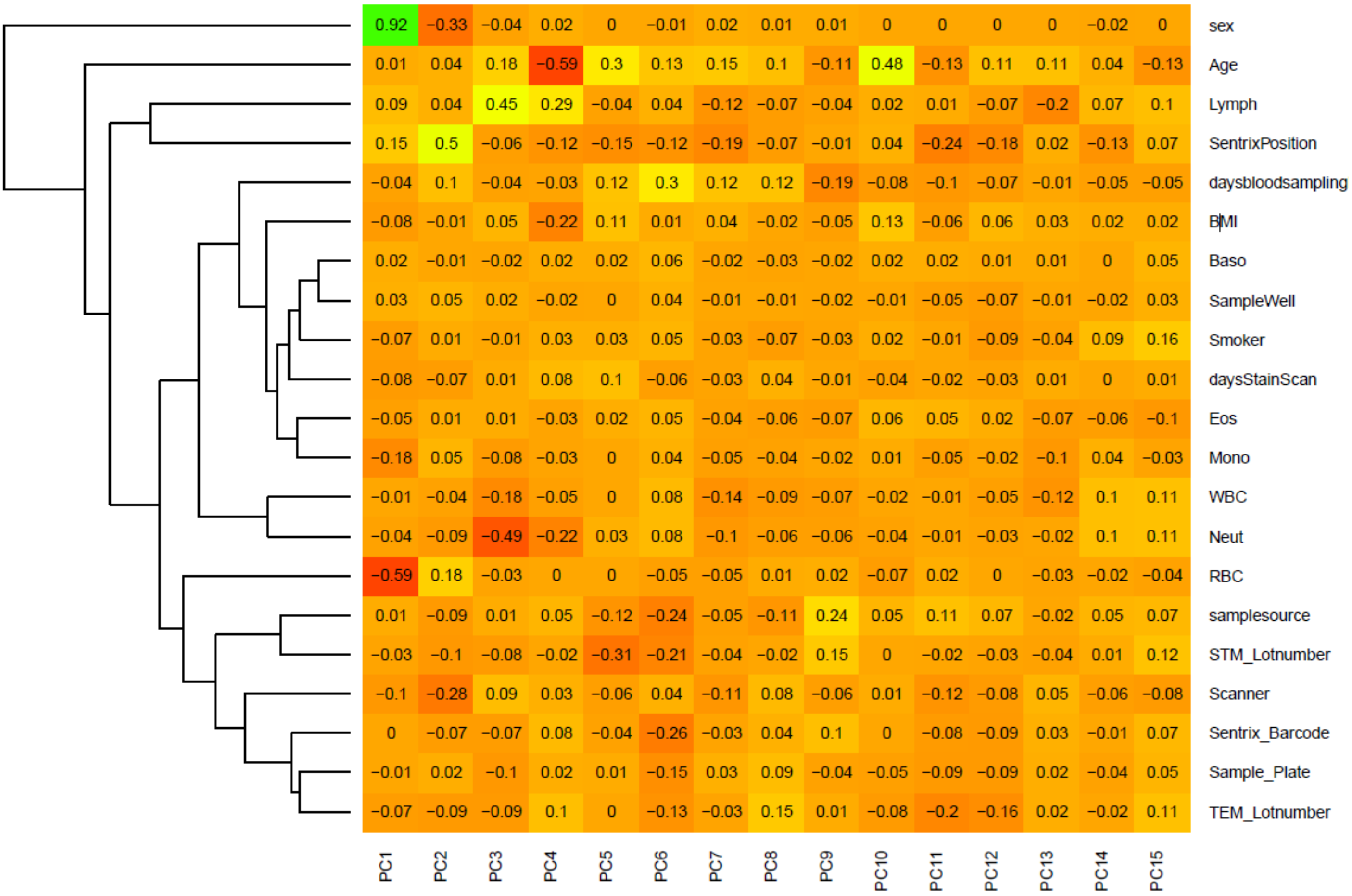

Supplementary Figure 26: Heatmap depicting the correlation between the first 15 Principal Components from the raw genome-wide methylation data (x-axis) and technical batches and biological effects (y-axis). Yellow-green= positive correlation. Orange-red= negative correlation. Lymph=lymphocyte counts. Daysbloodsmpling=Days between blood sampling and hybridization. BMI=Body Mass Index. Baso=Basophil count. daysStainScan=Days between staining and scanning. Eos=Eosinophil count. Mono=Monocyte count. WBC=Total white blood cell count. Neut=Neutrophil count. RBC=Red Blood cell count. Samplesource= 1 or 2, for individuals with 2 longitudinal samples (both from blood).

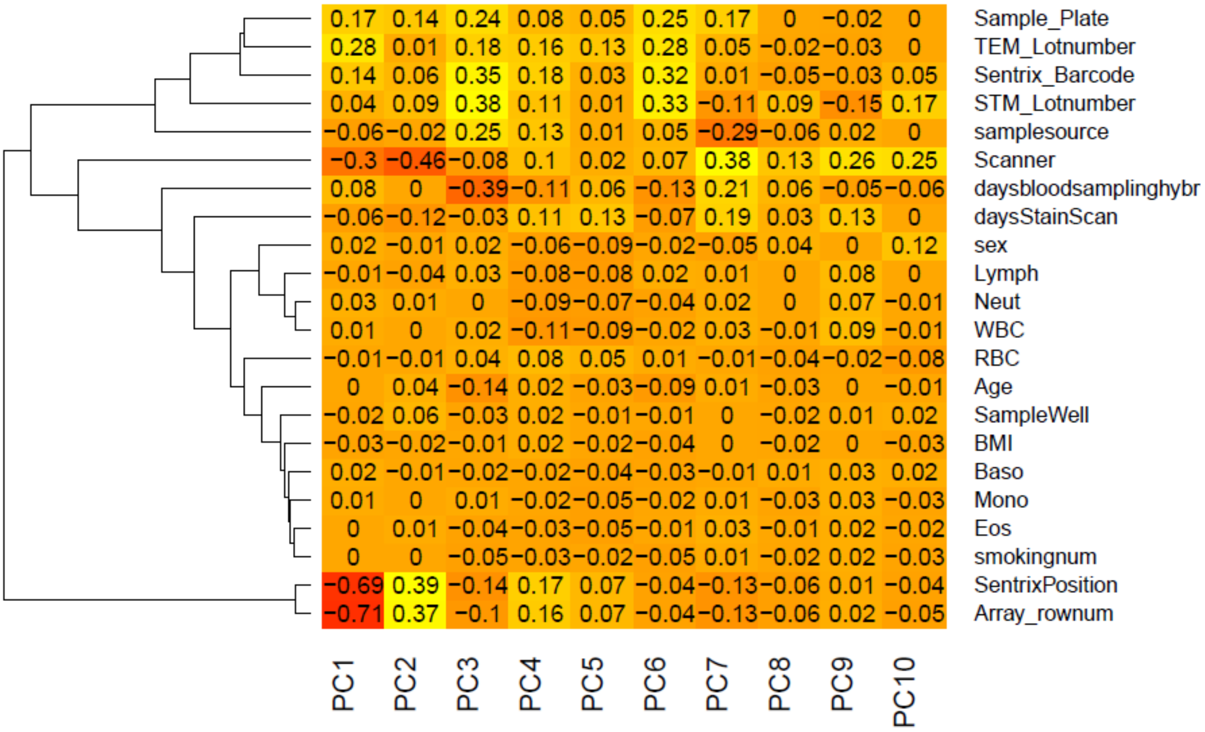

Supplementary Figure 27: Heatmap depicting the correlation between the first 10 Principal Components from the control probes (x-axis) and technical batches and biological variables (y-axis). Yellow-green= positive correlation. Orange-red=negative correlation. Lymph=lymphocyte counts. Daysbloodsamplinghybr=Days between blood sampling and hybridization. BMI=Body Mass Index. Baso=Basophil count. daysStainScan=Days between staining and scanning. Eos=Eosinophil count. Mono=Monocyte count. WBC=Total white blood cell count. Neut=Neutrophil count. RBC=Red Blood cell count. Samplesource= 1 or 2, for individuals with 2 longitudinal samples (both from blood). Array_rownum=Array row (numbered from 1 to 6).

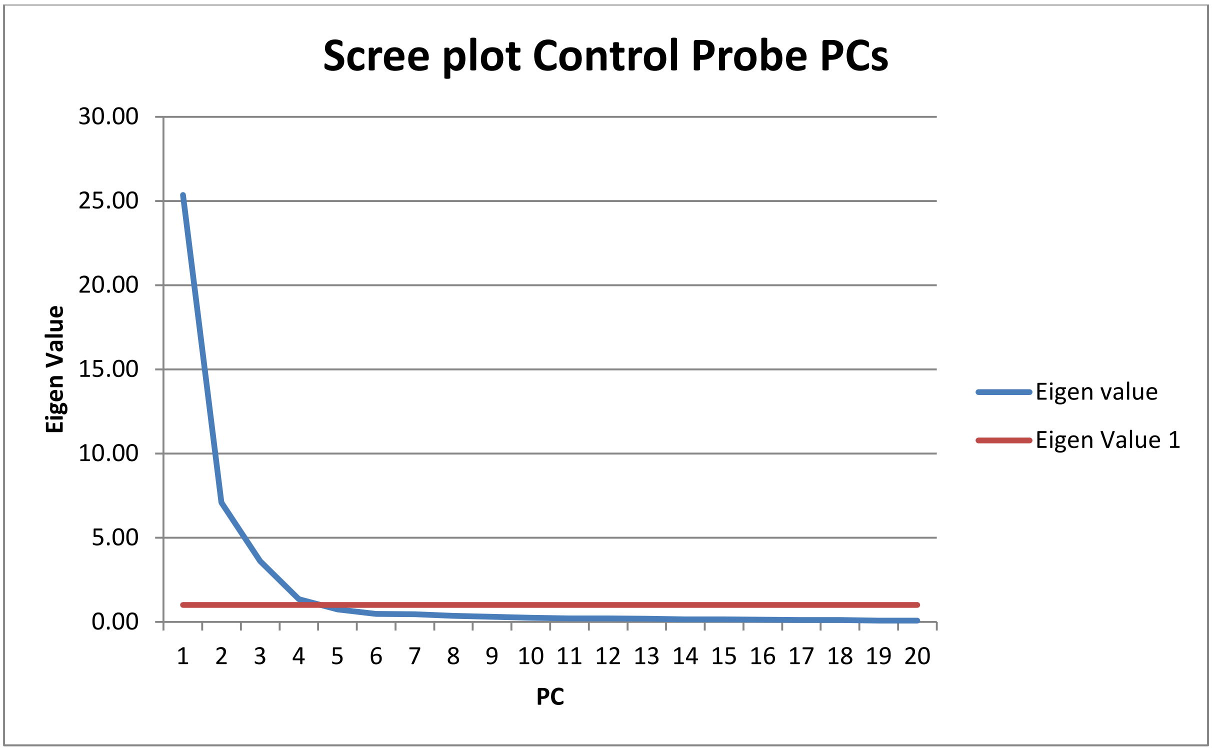

Supplementary Figure 28: Scree plot of the control probe PCs.

Supplementary Tables

Supplementary Table 1: Twin correlations and estimates of variance explained by A, C and D based on classical twin analyses of DNA methylation level at all autosomal CpGs.

Parameter	Min	Median	Mean	Max
*r*MZ	-0.14	0.12	0.20	0.99
*r*DZ	-0.25	0.06	0.09	0.89
ACE model				
a^2	0.00	0.09	0.18	0.98
c^2	0.00	0.00	0.03	0.82
e^2	0.00	0.88	0.80	1.00
ADE model				
a^2	0.00	0.05	0.13	0.96
d^2	0.00	0.01	0.08	0.95
e^2	0.00	0.87	0.79	1.00
AE model				
a^2	0.00	0.12	0.20	0.98
e^2	0.00	0.88	0.80	1.00

*r*MZ= Correlation between monozygotic twins. *r*DZ= Correlation between dizygotic twins.
a^2 =proportion of variance explained by additive genetic effects, c^2=proportion of variance explained by common environmental effects, e^2= proportion of variance explained by unique environmental effects, d^2 = proportion of variance explained by non-additive genetic effects. A= additive genetic effects, C= common environmental effects, D= non-additive genetic effects, E= unique environmental effects.

Supplementary Table 2: Twin correlations, heritability and longitudinal correlation, stratified by the amount of variation in DNA methylation between individuals.

		r MZ twins			*r* DZ twins			Classical Twin Heritability[A]			Longitudinal Correlation		
SD of the β-value	N CpGs	mean	SD	median	mean	SD	median	mean	SD	median	mean	SD	median
>= 0 (All)	411169	0.20	0.21	0.13	0.09	0.11	0.06	0.22	0.27	0.16	0.21	0.30	0.16
>= 0-0.01	49599	0.04	0.05	0.04	0.02	0.05	0.02	0.05	0.13	0.04	0.01	0.19	0.01
>= 0.01-0.02	171461	0.09	0.09	0.08	0.04	0.07	0.04	0.10	0.17	0.09	0.07	0.20	0.07
>= 0.02-0.03	85003	0.23	0.15	0.23	0.11	0.09	0.10	0.25	0.22	0.24	0.24	0.26	0.24
>= 0.03-0.04	49918	0.35	0.19	0.36	0.15	0.11	0.15	0.38	0.25	0.38	0.39	0.28	0.43
>= 0.04-0.05	27110	0.44	0.20	0.44	0.19	0.12	0.19	0.49	0.28	0.52	0.51	0.29	0.57
>= 0.05	28078	0.57	0.25	0.60	0.25	0.13	0.25	0.64	0.33	0.73	0.64	0.31	0.77

[A]Classical Twin heritability computed based on the twin correlations (see Methods).

SD= Standard deviation. β-value= Methylation beta-value, which represents the proportion of DNA Methylation. The first row summarizes the results for all analyzed CpGs and all other rows summarize the results for CpGs grouped based on the standard deviation of methylation level across all subjects.

Supplementary Table 3: Number of DNA methylation samples that failed sample quality checks.

**Quality Metric**	**N outliers[A]**
MU	47
BS	52
NP	44
HC	22
DP	48
**Combinations of failure**	**N outliers[B]**
BS	1
HC	14
HC + BS	5
HC + BS + MU	2
DP	3
DP + DS + MU	1
DP + NP + MU	1
DP + NP + MU + BS + MU	42
DP + NP + MU + BS + MU + HC	1
Total N bad quality samples[C]	70

The following five quality metrics were computed with the R package MethylAid: **MU**= median Methylated versus Unmethylated signal intensity, **BS**=Efficiency of bisulphite conversion, **NP**=overall quality based on sample-dependent control probes (non-polymorphic quality control probes), **HC**= overall quality based on sample-independent hybridization control probes. **DP**= Fraction of probes per sample where the signal exceeds the background signal, as assessed with the detection p-value, which uses the negative control probes to assess background signal.

[A]N outliers= Number of samples that failed based on each quality metric.

[B]N outliers= Number of samples that failed based on a particular combination of multiple quality metrics.

[C]All samples that failed based on one or more of the five quality metrics were discarded (70 samples).

Supplementary Table 4: Eigen values and proportion of variance explained by Principle Components 1 to 15, calculated based on the raw genome-wide methylation data.

	**PC1**	**PC2**	**PC3**	**PC4**	**PC5**	**PC6**	**PC7**	**PC8**	**PC9**	**PC10**	**PC11**	**PC12**	**PC13**	**PC14**	**PC15**
Eigen value	54.71	37.13	14.09	5.65	3.95	2.71	1.51	1.23	0.98	0.95	0.68	0.65	0.55	0.49	0.46
Proportion of Variance	0.2211	0.1501	0.0569	0.0228	0.0160	0.0110	0.0061	0.0050	0.0040	0.0038	0.0028	0.0026	0.0022	0.0020	0.0019
Cumulative Proportion	0.2211	0.3712	0.4281	0.4510	0.4669	0.4779	0.4840	0.4890	0.4929	0.4968	0.4995	0.5021	0.5044	0.5063	0.5082

Supplementary Table 5: Eigen values and proportion of variance explained by Principle Components 1 to 10, calculated based on control probes from the methylation array.

	**PC1**	**PC2**	**PC3**	**PC4**	**PC5**	**PC6**	**PC7**	**PC8**	**PC9**	**PC10**
Eigen value	25.37	7.09	3.61	1.36	0.73	0.48	0.45	0.36	0.30	0.25
Proportion of Variance	0.6039	0.1687	0.0860	0.0323	0.0175	0.0114	0.0108	0.0087	0.0071	0.0060
Cumulative Proportion	0.6039	0.7727	0.8587	0.8910	0.9085	0.9199	0.9307	0.9393	0.9465	0.9524
